# BGN/MDK Axis in the Melanoma Tumor Microenvironment Strengthens Tumor Malignancy by Modulating Cancer Cells and Cancer‐Associated Fibroblasts Crosstalk

**DOI:** 10.1002/advs.202514590

**Published:** 2026-03-15

**Authors:** Hao‐ze Shi, Ming‐yang Wu, Jin‐quan Liu, Cuicui Tian, Li Ma, Xue‐mei Zhou, Ze‐Hao Sun, Zhi‐yong Xu, Run‐dong Zhang, Shan‐yuan Ye, Li‐ming Huang, Yan Wang, Jing‐shu Xiong, Wen‐bo Bu, Xian‐feng Cheng, Jian‐fang Sun, Hao Chen

**Affiliations:** ^1^ Hospital for Skin Diseases Institute of Dermatology Chinese Academy of Medical Sciences & Peking Union Medical College Nanjing Jiangsu China; ^2^ Department of Dermatology Shenzhen People's Hospital (The Second Clinical Medical College Jinan University The First Affiliated Hospital Southern University of Science and Technology) Shenzhen Guangdong China; ^3^ Jiangsu Cancer Hospital and The Affiliated Cancer Hospital of Nanjing Medical University Nanjing Jiangsu China; ^4^ Weifang People's Hospital The First Affiliated Hospital of Shandong Second Medical University Weifang Shandong China

**Keywords:** biglycan (BGN), cancer‐associated fibroblasts (CAFs), melanoma, midkine (MDK), tumor microenvironment

## Abstract

While the function of biglycan (BGN) is recognized in various cancers, its precise role and the mechanisms underlying cancer‐associated fibroblasts (CAFs) formation within the melanoma tumor microenvironment (TME) remain poorly understood. Utilizing spatial transcriptomics, single‐cell RNA sequencing (scRNA‐seq), vitro/vivo assays, function analysis and molecular assays, this study comprehensively investigated the BGN regulatory network. We discovered that N6‐methyladenosine (m6A) modulators—specifically YTHDF3, YTHDC1, and METTL14—cooperatively upregulate BGN expression in a parallel, non‐hierarchical manner converging on functional m6A sites within melanoma cells. Consequently, BGN significantly promoted melanoma proliferation and metastasis. Within the TME, spatial transcriptomics and scRNA‐seq revealed that CAFs, rather than tumor cells, exhibited the highest BGN expression. Cell trajectory analysis indicated that myCAFs^BGN‐high^ may originate from iCAFs^BGN‐low^ to interact with melanoma cells. Furthermore, midkine (MDK) signaling pathways was identified by cell chat analysis. Transcriptomic and spatial analysis revealed that BGN could regulate its expression at RNA and protein levels in CAFs through the regulator AE binding protein 1 (AEBP1). And the tumor promotion effect of CAFs may be executed by BGN/MDK axis, which also reduced CD8⁺ T cell infiltration in TME. Pharmacological inhibition of MDK also suppressed tumor growth with increased CD8⁺ T cell infiltration. Finally, this BGN/MDK axis could also drive the activation of normal fibroblasts into a CAF‐like phenotype verified by vitro assays. In conclusion, the interplay between cancer cells and CAFs mediated by the BGN/MDK axis is a critical driver of malignancy in melanoma, highlighting it as a promising therapeutic target for intervention.

## Graphical abstract

This study reveals that m6A regulators cooperatively upregulate BGN in melanoma, promoting malignancy. Within the tumor microenvironment, CAFs show highest BGN expression. The BGN/MDK axis mediates cancer‐stroma crosstalk, driving normal fibroblast (NF) activation and enhancing the pro‐tumor effect of CAFs, highlighting a promising therapeutic target.

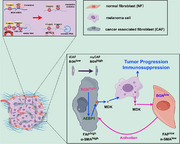

**Abbreviations**
AEBP1: AE binding protein 1
BCC: basal cell carcinoma
BGN: biglycan
CAFLCs: cancer‐associated fibroblast‐like cells
CAFs: cancer‐associated fibroblasts
CM: culture media
cSCC: cutaneous squamous cell carcinoma
CS/DS: chondroitin sulfate/dermatan sulfate
CXCLs: chemokine ligands
ECM: extracellular matrix
EdU: 5‐Ethynyl‐2′‐deoxyuridine
ELISA: enzyme‐linked immunosorbent assay
ER: estrogen receptor
ESCC: esophageal squamous cell carcinoma
FBS: fetal bovine serum
FGF: fibroblast growth factors
GAG: glycosaminoglycan
H&E: haematoxylin and eosin
iCAFs: inflammatory CAFs
IHC: immunohistochemistry
IL‐1: interleukin‐1
IL‐6: interleukin‐6
m^6^A: N6‐methyladenosine
MC: mesothelial cell
MDK: midkine
mIHC: multiplex immunohistochemical
myCAFs: myofibroblastic CAFs
NFs: normal fibroblasts
OD: optical density
RBPs: RNA‐binding proteins
scRNA‐seq: single‐cell RNA sequencing
TGF‐β: transforming growth factor‐β
TME: tumor microenvironment
TNBC: triple‐negative breast cancer

## 1 Introduction

As a prevalent RNA methylation manner in eukaryotic cells, N^6^‐methyladenosine (m^6^A) commonly modifies messenger RNA molecules [1]. The modification of m^6^A may influence several processes, including RNA splicing, decay, export, and translation [1]. Multiple m^6^A‐associated proteins have been validated as contributors to tumor development, with particularly prominent roles observed in melanoma pathogenesis [2, 3, 4]. In our earlier research, we found that YTHDF3 could independently participate in the translation initiation of its target mRNAs, such as LOXL3, and contribute to tumor progression [4]. Further analysis of our previous study also found that in melanoma A375 cells, the biglycan (BGN) mRNA contains multiple m^6^A sites, which can be bound by YTHDF3. Upon intervention of YTHDF3 expression, the expression levels of BGN mRNA and protein are altered. Our research indicates that higher levels of BGN expression in melanoma cells can enhance their malignant biological behavior.

In accordance with the “seed and soil” theory of cancer metastasis, metastatic tumor cells depend on an appropriate tumor microenvironment (TME), and they have the ability to alter the function of stromal cells within the environment they colonize [5]. By releasing different cytokines like transforming growth factor‐β (TGF‐β), fibroblast growth factors (FGF), interleukin‐1 (IL‐1), and chemokine ligands (CXCLs), tumor cells can induce normal fibroblasts (NFs) to become cancer‐associated fibroblasts (CAFs) [6]. In the precancerous lesions of breast cancer, the epithelial–mesenchymal transition mediated by Jagged1/Notch2 ligand–receptor interaction could activate the conversion of NFs into CAFs [7]. These studies suggested that tumor cells may mediate the functional activation of fibroblasts.

CAFs have been identified as a vital element within the TME, significantly influencing both cancer progression and therapy response [8]. In skin cancer, particularly melanoma, CAFs demonstrated significant heterogeneity and functional diversity, which could play an essential role in tumor biology and patient outcomes [9, 10, 11, 12, 13]. Traditionally viewed as mere structural cells, fibroblasts are now acknowledged for its plasticity and functional variety, particularly in pathological conditions. They not only participate in the deposition of extracellular matrix (ECM) but also regulate various cellular processes within their microenvironment [14]. Recent research indicated that fibroblasts could contribute to cancer development, underscoring their role in tumorigenesis. Via scRNA‐seq, it has discovered that different subpopulations of dermal fibroblasts was enriched in healthy skin across both mice and humans [15].

Fibroblast heterogeneity is evident in skin cancers like basal cell carcinoma (BCC) [9], melanoma or cutaneous squamous cell carcinoma (cSCC) [12]. CAFs comprise two principal subtypes: myofibroblastic CAFs (myCAFs) and inflammatory CAFs (iCAFs), distinguished by their functional phenotypes, exhibiting unique functions and marker gene expressions. For instance, myCAFs are distinguished by elevated levels of ECM components [16, 17] and myCAFs could facilitating tumor suppression by leading T cell exclusion [18]. In addition, iCAFs can engage with different immune cells like CD8^+^ T cells, potentially fostering an immunosuppressive tumor microenvironment [19]. Furthermore, some CAFs could directly interact with tumor cells, enhancing their ability of survival, proliferation, and migration [20, 21]. These CAFs could also influence the behavior of other stromal cells, such as endothelial cells, to promote tumor angiogenesis [22].

Melanoma, which originates from melanocytes, is characterized by its high metastatic potential and low survival rates. CAFs in melanoma have been implicated in tumor progression and immune modulation, with the iCAF subtype being particularly associated with aggressive tumors, showing increased abundance in advanced stage melanoma [10]. These CAFs could secrete numerous immunomodulatory factors that interact with receptors on neutrophils, T cells, and NK cells, thereby affecting immune cell recruitment and activation [10]. A comprehensive understanding of CAFs heterogeneity and functionality in melanoma has significant clinical implications, diverse functions of CAFs position them as promising targets for therapeutic strategies. Continued investigation into the molecular mechanisms driving CAFs heterogeneity and for cancer treatment advancements, their interactions with other TME cellular elements are essential.

In this study, we discovered that several m^6^A modulators like YTHDF3, YTHDC1, and METTL14 could cooperatively regulate BGN expression in a parallel, nonhierarchical manner, delay RNA degradation or affect the translation process of BGN via eIF3A. The regulatory function of these molecules depends on m^6^A modifications on the BGN transcript and enhanced expression of BGN could promote the malignancy of melanoma. Through spatial transcriptomics and scRNA‐seq analysis, it was found that CAFs in the melanoma microenvironment, had the highest levels of BGN expression. Among these CAFs, myCAFs had higher expression level of BGN compared with iCAFs, and myCAFs had a more robust connection with tumor cells. Mechanistically, BGN upregulates the key secreted factor midkine (MDK) in CAFs, partly through the transcriptional regulator AEBP1. Finally, the BGN/MDK axis in CAFs drives melanoma progression and may influence CD8^+^ T cell infiltration in TME. Reciprocally, the activation of this axis within melanoma cells can stimulate the activation of normal fibroblasts toward a CAF‐like phenotype, ultimately reinforcing a protumorigenic feedback loop.

## 2 Results

### 2.1 The Regulation of BGN in Melanoma Cells Depends on m^6^A Modulators

**FIGURE 1:**
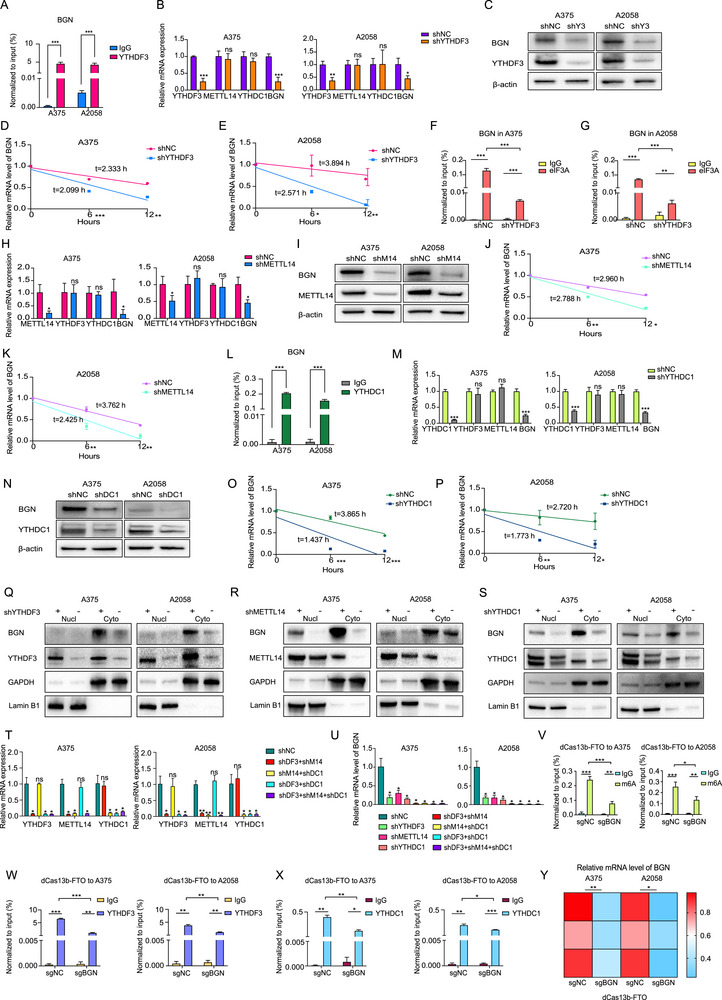
The expression of BGN is regulated by m^6^A modification associated proteins in melanoma. (A) RIP‐qPCR shows the interaction between YTHDF3 protein and mRNA of BGN in A375 and A2058 melanoma cells. (B) mRNA level of YTHDF3, METTL14, YTHDC1, and BGN after downregulation of YTHDF3 by RT‐qPCR in A375 and A2058 melanoma cells. (C) Protein level of YTHDF3, METTL14, YTHDC1, and BGN after downregulation of YTHDF3 by western blot in A375 and A2058 melanoma cells. (D,E) The mRNA stability of BGN at different time points after actinomycin D (Act D, 10 mg/mL) treatment in A375 and A2058 cells after YTHDF3 downregulation. (F,G) RIP‐qPCR shows the interaction between eIF3A protein and mRNA of BGN in shNC and shYTHDF3 of A375 and A2058 cells. (H) mRNA level of METTL14, YTHDF3, YTHDC1, and BGN after downregulation of METTL14 by RT‐qPCR in A375 and A2058 melanoma cells. (I) Protein level of METTL14, YTHDF3, YTHDC1, and BGN after downregulation of METTL14 by western blot in A375 and A2058 melanoma cells. (J,K) The mRNA stability of BGN at different time points after actinomycin D (Act D, 10 mg/mL) treatment in A375 and A2058 cells after METTL14 downregulation. (L) RIP‐qPCR shows the interaction between YTHDC1 protein and mRNA of BGN in A375 and A2058 melanoma cells. (M) mRNA level of YTHDC1, YTHDF3, METTL14, and BGN after downregulation of YTHDC1 by RT‐qPCR in A375 and A2058 melanoma cells. (N) Protein level of YTHDC1, YTHDF3, METTL14, and BGN after downregulation of YTHDC1 by western blot in A375 and A2058 melanoma cells. (O,P) The mRNA stability of BGN at different time points after actinomycin D (Act D, 10 mg/mL) treatment in A375 and A2058 cells after YTHDC1 downregulation. (Q) Protein level of BGN in nucleus and cytoplasm after downregulation of YTHDF3 by western blot in A375 and A2058 melanoma cells. (R) Protein level of BGN in nucleus and cytoplasm after downregulation of METTL14 by western blot in A375 and A2058 melanoma cells. (S) Protein level of BGN in nucleus and cytoplasm after downregulation of YTHDC1 by western blot in A375 and A2058 melanoma cells. (T) mRNA levels of YTHDF3, METTL14, YTHDC1 after combinatorial or triple downregulation of the indicated regulators in A375 and A2058 melanoma cells measured by RT‐qPCR. (U) mRNA levels of BGN after individual, combinatorial or triple downregulation of YTHDF3, METTL14, YTHDC1 in A375 and A2058 melanoma cells measured by RT‐qPCR. (V) m^6^A level of dCas13b‐FTO‐sgNC group and dCas13b‐FTO‐sgBGN group detected by MeRIP‐qPCR in A375 and A2058 melanoma cells. (W) Interaction between YTHDF3 protein and BGN mRNA in groups of dCas13b‐FTO‐sgNC and dCas13b‐FTO‐sgBGN measured by RIP‐qPCR in A375 and A2058 melanoma cells. (X) Interaction between YTHDC1 protein and BGN mRNA in groups of dCas13b‐FTO‐sgNC and dCas13b‐FTO‐sgBGN measured by RIP‐qPCR in A375 and A2058 melanoma cells. (Y) mRNA levels of BGN in dCas13b‐FTO‐sgNC group and dCas13b‐FTO‐sgBGN group measured by RT‐qPCR. Data are shown as means ± S.D. *, **, and *** means *p* < 0.05, *p* < 0.01, and *p* < 0.001, respectively.

According to our previous research [4], we observed that reducing YTHDF3 expression in melanoma A375 cells could also lead to a decrease in BGN RNA expression levels and BGN mRNA could be captured by YTHDF3. Consequently, we employed RIP‐qPCR and observed that YTHDF3 was able to capture BGN mRNA in melanoma cells, including A375 and A2058 (Figure 1A). Our earlier studies indicated that YTHDF3 can control melanoma metastasis through an m^6^A‐dependent mechanism [4], and we also wonder if some m^6^A regulators, which were not reported in melanoma, such as METTL14 and YTHDC1, could also participate in the expression of BGN. In melanoma cells such as A375 and A2058, our research revealed that the mRNA (Figure 1B,H,M) and protein level (Figure 1C,I,N) of BGN were impacted following the downregulation of YTHDF3, METTL14, and YTHDC1. Yet, these three molecules were almost not interacted on each other both at RNA (Figure 1B,H,M) and protein level (Figure S1A–F). BGN mRNA could also be captured by YTHDC1, a kind of m^6^A nuclear reader (Figure 1L). We noticed that the regulation could be both occurred in nucleus and cytoplasm (Figure 1Q,R,S). After the downregulation of these three regulators, the RNA stability of BGN was decreased (Figure 1D,E,J,K,O,P). Our prior findings indicated that YTHDF3 can modulate the translation initiation of its targets using eIF3A. In this study, we also observed that eIF3A was not enriched in BGN mRNA following YTHDF3 downregulation (Figure 1F,G).

To delineate the interactive regulatory mode among above factors, we performed combinatorial knockdown experiments in melanoma cells including A375 and A2058. The mRNA and protein expression of each regulator was not affected by the knockdown dual knockdowns of the others (e.g., METTL14/YTHDF3, METTL14/YTHDC1, YTHDF3/YTHDC1) (Figure 1T; Figure S1G,H). While individual knockdown of YTHDF3, METTL14, or YTHDC1 reduced BGN expression, dual knockdowns and the triple knockdown resulted in a more pronounced reduction in both BGN mRNA and protein levels (Figure 1U; Figure S1G,H). Notably, the inhibitory effects observed in the dual‐knockdown groups were comparable to that of the triple‐knockdown group. The above results suggest a parallel, nonhierarchical cooperative relationship rather than a simple linear pathway. Besides, considering their distinct subcellular localizations, this implies that YTHDF3, METTL14, and YTHDC1, and likely exert independent yet coordinated control over BGN within their respective nuclear or cytoplasmic compartments, converging on the same m^6^A‐modified transcript to ensure regulation.

Furthermore, to obtain direct evidence that the observed regulation is mediated by m^6^A modifications on BGN mRNA itself, we employed a dCas13b‐FTO fusion system for specific demethylation in melanoma cells A375 and A2058. Guided by a specific sgRNA (sgBGN), the dCas13b‐FTO complex significantly reduced the m^6^A level on BGN transcripts, as confirmed by MeRIP‐qPCR (Figure 1V). This targeted m^6^A erasure abolished the binding of both YTHDF3 and YTHDC1 to BGN mRNA confirmed by RIP‐qPCR (Figure 1W,X), and led to a slight decrease in BGN expression both in RNA (Figure 1Y) and protein level (Figure S1I). These experiments provide evidence that the m^6^A modification on BGN is required for reader recognition and for maintaining optimal expression levels.

Collectively, m^6^A regulators (YTHDF3, METTL14, YTHDC1) posttranscriptionally modulate BGN expression at both RNA and protein levels in melanoma cells.

### 2.2 BGN Drives Melanoma Malignancy In Vitro

**FIGURE 2:**
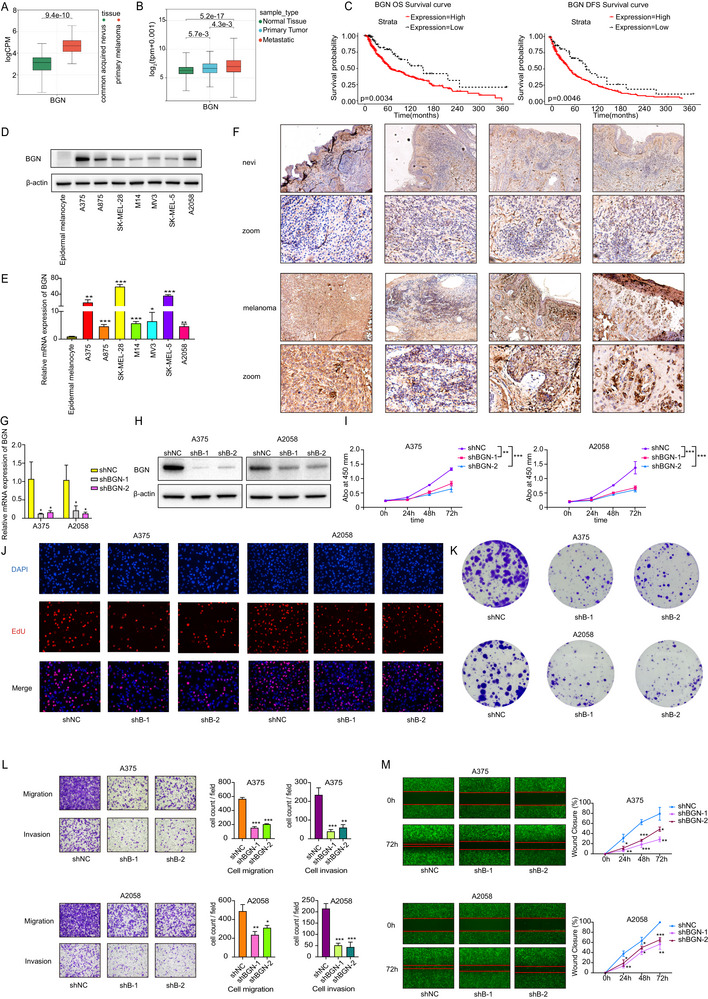
The expression of BGN in melanoma and downregulation of BGN inhibits melanoma progression in vitro. (A) mRNA expression of BGN in nevus tissues (left, green bar) and melanoma tissues (right, red bar) in GSE98394 dataset. (B) mRNA expression of BGN in normal tissues (left, green bar), primary melanoma tissues (middle, blue bar), and metastatic melanoma tissues (right, red bar) in TCGA dataset and GTEx dataset. (C) The survival condition of melanoma patients with different expression level of BGN in TCGA dataset and GTEx dataset. (D) BGN protein level in epidermal melanocyte and melanoma cell lines (A375, A875, SK‐MEL‐28, M14, MV3, SK‐MEL‐5, and A2058) verified by western blot. (E) BGN mRNA level in epidermal melanocyte and melanoma cell lines (A375, A875, SK‐MEL‐28, M14, MV3, SK‐MEL‐5, and A2058) verified by RT‐qPCR. (F) BGN protein level in melanoma tissues and nevi tissues assessed by immunohistochemistry. Scale bar = 100 or 20µm. (G,H) A375 and A2058 cells are transfected with two shRNAs targeting BGN and a negative control shRNA. Expression of BGN mRNA and protein are detected by RT‐qPCR and western blot. (I–K) CCK‐8, EdU assay, and clone formation assay show that BGN downregulation inhibits proliferation ability of A375 and A2058 melanoma cells. (L,M) Transwell assay and wound healing assay show that BGN downregulation inhibits migration and invasion ability of A375 and A2058 melanoma cells in 24, 48, and 72 h. Data are shown as means ± S.D. *, **, and *** means *p* < 0.05, *p* < 0.01, and *p* < 0.001, respectively.

**FIGURE 3:**
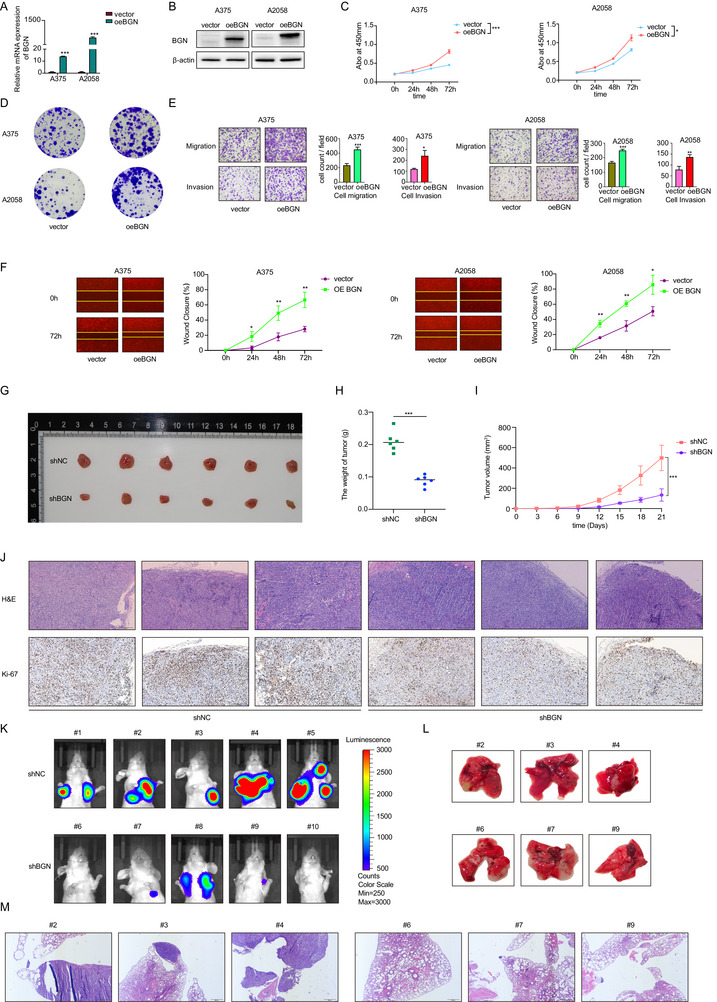
Upregulation of BGN promotes melanoma progression in vitro and downregulation of BGN inhibits melanoma progression in vivo. (A,B) A375 and A2058 cells are transfected with lentivirus overexpressing BGN and a blank vector. Expression of BGN mRNA and protein are detected by RT‐qPCR and western blot. (C,D) CCK‐8 and clone formation assay show that BGN upregulation promotes proliferation ability of A375 and A2058 melanoma cells. (E,F) Transwell assay and wound healing assay show that BGN upregulation promotes migration and invasion ability of A375 and A2058 melanoma cells in 24, 48, and 72 h. (G–J) BGN downregulation inhibits subcutaneous tumor proliferation of A375 melanoma cells in mice. Scale bar = 100 µm. (K–M) BGN downregulation inhibits lung metastasis of A375 melanoma cells in mice. Scale bar = 200 µm. Data are shown as means ± S.D. *, **, and *** means *p* < 0.05, *p* < 0.01, and *p* < 0.001, respectively.

Bioinformatic and clinical analyses revealed BGN upregulation in melanoma (notably metastatic lesions), correlating with poorer patient survival (Figure 2A–C). Compared with epidermal melanocyte and nevi tissue samples, there was a significantly increased expression of BGN in both melanoma cell lines and tissue samples (Figure 2D–F). Then, we constructed BGN downregulation (Figure 2G,H) and overexpression model (Figure 3A,B) in A375 and A2058 cells. Results from the CCK‐8 (Figures 2I and 3C), EdU assay (Figure 2J), and clone formation assay (Figures 2K and 3D) demonstrated a positive correlation between BGN and the growth ability of melanoma cells. Our findings also indicated that BGN was positively associated with the migration and invasion abilities of melanoma cells, as shown in the transwell assay (Figures 2L and 3E) and the wound healing assay (Figures 2M and 3F). Therefore, we demonstrated that BGN could regulate the malignancy of melanoma cells.

### 2.3 BGN Regulates the Malignant Behaviors of Melanoma In Vivo

Using nude mice, we established the subcutaneous tumor models and tail vein injection metastatic tumor models. The A375 cells with BGN downregulation (shBGN) and the control shNC cells were administered to nude mice. We observed that, at 3 and 6 weeks, mice injected with cells where BGN was downregulated had smaller subcutaneous tumors (Figure 3G–I), a lower proliferation rate (Figure 3J), and reduced lung metastasis (Figure 3K–M). Collectively, our results showed that BGN could govern melanoma metastasis in vivo.

### 2.4 BGN Positive Cancer Associated Fibroblasts Scattered Around the Melanoma Mass

**FIGURE 4:**
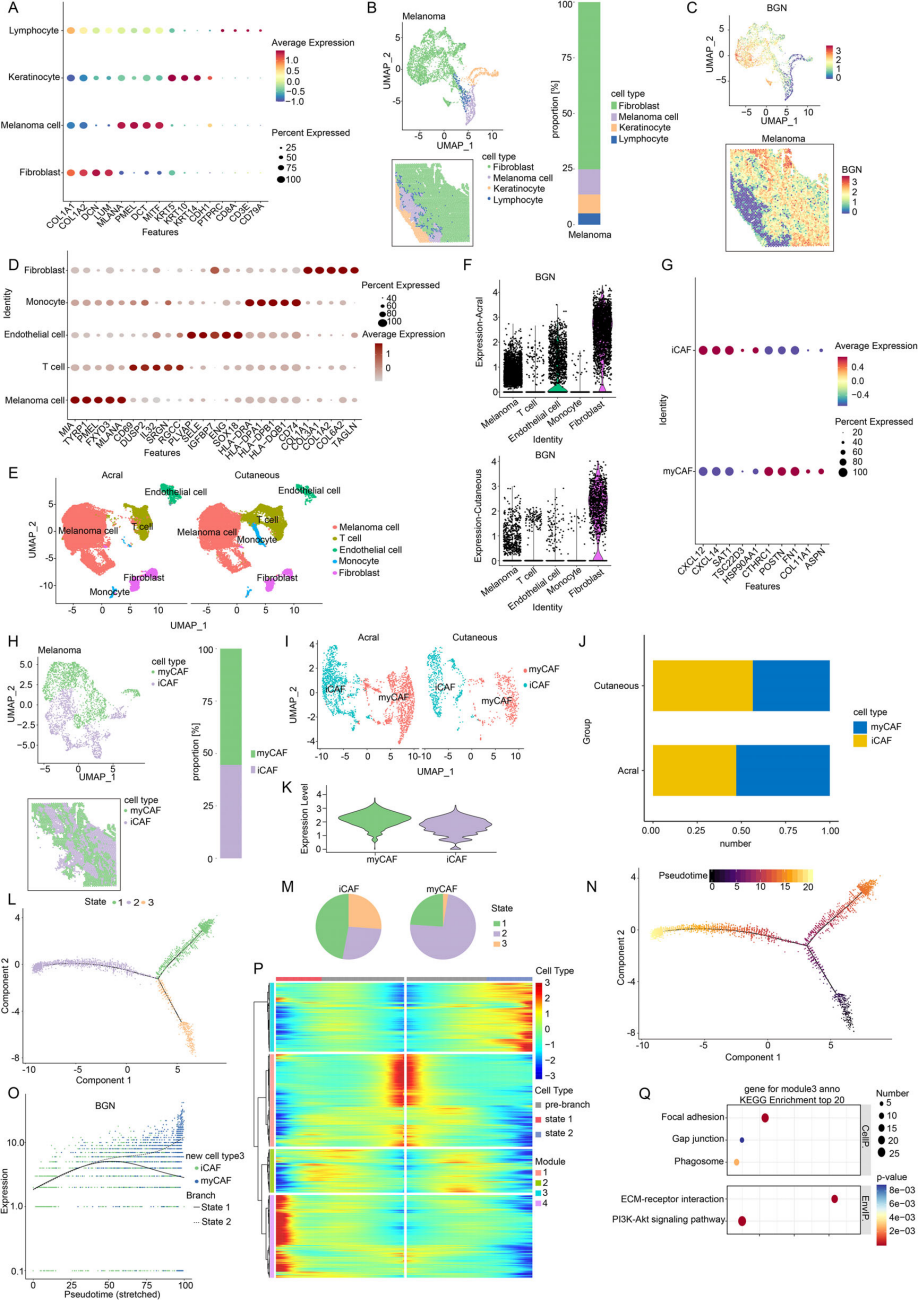
The expression of BGN in CAFs in melanoma tumor microenvironment and its role in CAFs evolution. (A,B) Four cluster cells including melanoma cell, fibroblast, keratinocyte and lymphocyte are identified from results of spatial transcriptome. (C) Expression of BGN from the results of spatial transcriptome. (D,E) Cell types identified from published scRNA‐seq data of melanoma tissues including acral melanoma and cutaneous melanoma. (F) Expression of BGN from the results of published scRNA data. (G) Markers used to identify myCAFs and iCAFs. (H) Condition of myCAFs and iCAFs from the results of spatial transcriptome. (I,J) Condition of myCAFs and iCAFs from the results of published scRNA‐seq data. (K) Expression of BGN in myCAFs and iCAFs from the results of spatial transcriptome. (L–N) Cell trajectory analysis to find the relationship between myCAFs and iCAFs. (O) The expression level of BGN during iCAFs evolution. (P) The heat map of differentially expressed genes during iCAFs evolution. (Q) The terms of KEGG analysis of module 3 in heat map.

Given the potent protumorigenic role of tumor cell‐intrinsic BGN established above, we sought to understand its broader relevance in the melanoma tumor microenvironment. Clinical and pathological observations suggest that tumor progression is not solely governed by cancer cell‐autonomous mechanisms but is profoundly shaped by the tumor microenvironment. Notably, our preliminary examination of melanoma tissue sections indicated that BGN expression was not confined to tumor cells but was also prominent in stromal cells. This observation prompted us to hypothesize that BGN might also function within the TME. Therefore, we next investigated the expression pattern and potential role of BGN in the stromal compartment of melanoma. First, a melanoma tissue was applied to the analysis of spatial transcriptome. Four cluster cells were identified, including melanoma cell, fibroblast, keratinocyte and lymphocyte (Figure 4A,B). And we noticed that BGN has relatively higher expression in the spots of fibroblast (Figure 4C). Next, we analyzed the published scRNA‐seq data of melanoma tissues including acral melanoma and cutaneous melanoma (Figure 4D,E). We also noticed that BGN had a relatively higher expression in fibroblasts both in acral melanoma and cutaneous melanoma (Figure 4F; Figure S2A,B). BGN regulatory mechanisms in melanoma were probed via GO/KEGG pathway analysis of downstream DEGs in fibroblasts. Results revealed predominant enrichment in extracellular matrix organization and cell communication pathways (Figure S2C,D). Subsequently, we supposed these cells as the BGN positive CAFs. Since myCAFs and iCAFs were the main component of CAFs, we identified these cells from CAFs in the analysis of spatial transcriptome. Markers like CXCL12, CXCL14, SAT1, TSC22D3, and HSP90AA1 were used to identify iCAFs, then CTHRC1, POSTN, FN1, COL11A1, and ASPN for myCAFs (Figure 4G). We found that iCAFs and myCAFs had approximately same number from the results of spatial transcriptome (Figure 4H) and published scRNA sequencing data (Figure 4I,J). Besides, compared with iCAFs, it seems that myCAFs had a slight relatively higher expression level of BGN (Figure 4K). Previous reports showed that iCAFs may be involved into myCAFs, so we applied cell trajectory analysis to find the relationship between these two cell groups. The results showed that cells of state 1 and 3 were the main components of iCAFs and state 2 was the main component of myCAFs (Figure 4L–N). Although stage 3 was also the component of myCAFs, yet its proportion was quite low. We set the stage 3 as the origin and other two stages as the endpoints (Figure 4L–N). From stage 3 to stage 2, the expression level of BGN was gradually increased, while it was nearly not changed during the process from stage 3 to stage 1 (Figure 4O). Besides, the heat map of differentially expressed genes showed that the module 3 was consisted of the differentially higher expressed genes of stage 2 (Figure 4P). The terms of GO analysis and KEGG analysis of this module were mainly with ECM related process, which hinted the clues of the transformation of myCAFs (Figure S3A–C; Figure 4Q). Therefore, we supposed that BGN may be associated with the conversion between iCAFs and myCAFs, especially in the final stage of CAFs (myCAFs).

### 2.5 BGN Positive Cancer Associated Fibroblasts Drives Melanoma Malignancy In Vitro/Vivo

**FIGURE 5:**
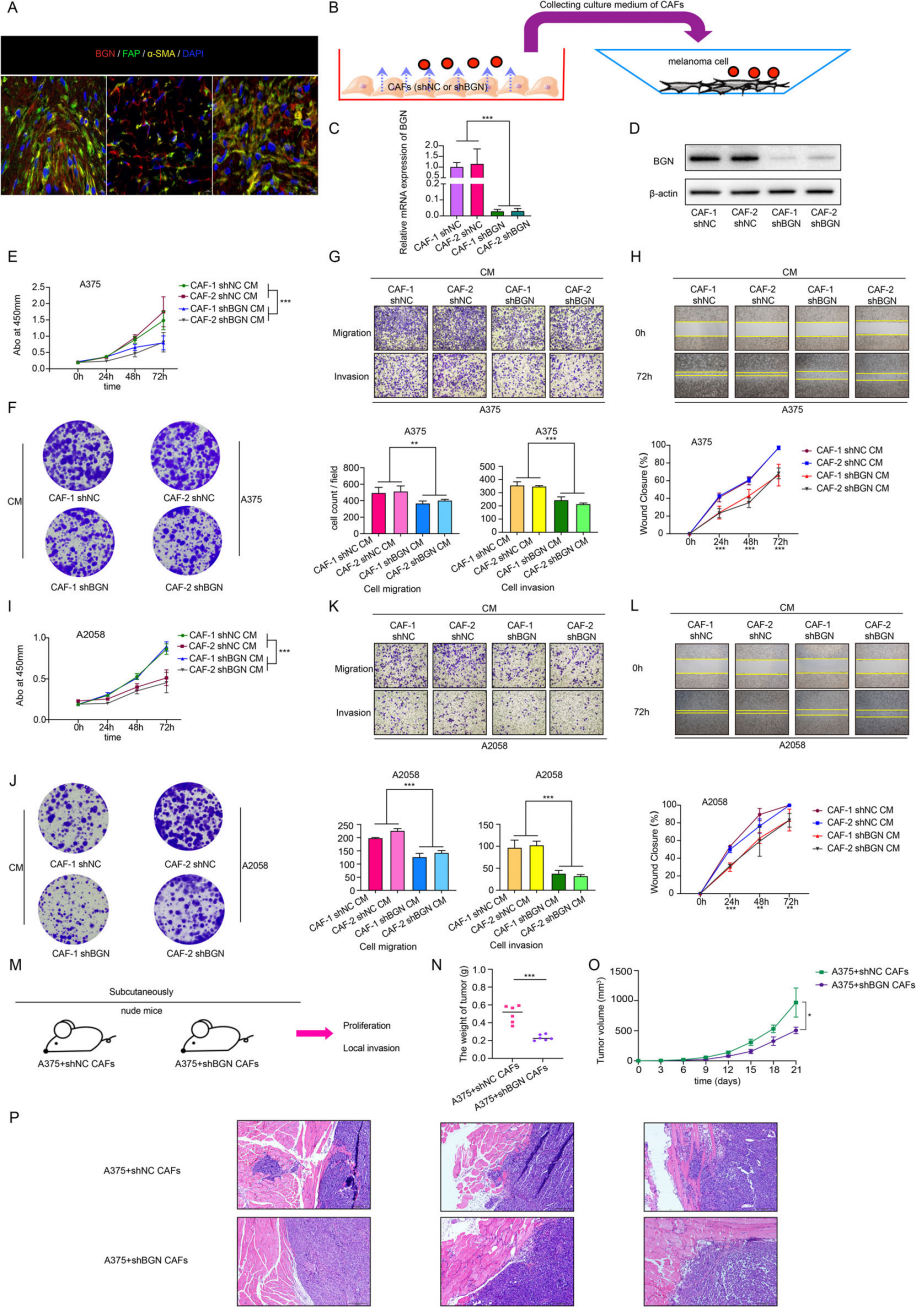
BGN in CAFs plays an oncogenic role in melanoma progression. (A) CAFs identified in three melanoma tissues by mIHC. Scale bar = 12.5µm. (B) The schematic diagram of culture model. (C,D) CAFs from different samples are transfected with shRNAs targeting BGN and a negative control shRNA. Expression of BGN mRNA and protein are detected by RT‐qPCR and western blot. (E,F) CCK‐8 assay and clone formation assay show that BGN in CAFs derived culture medium promotes proliferation ability of A375 melanoma cells. (G,H) Transwell assay and wound healing assay show that BGN in CAFs derived culture medium promotes migration and invasion ability of A375 melanoma cells. (I,J) CCK‐8 assay and clone formation assay show that BGN in CAFs derived culture medium promotes proliferation ability of A2058 melanoma cells. (K,L) Transwell assay and wound healing assay show that BGN in CAFs derived culture medium promotes migration and invasion ability of A2058 melanoma cells. (M) The schematic diagram of in vivo model. (N,O) BGN in CAFs promote subcutaneous tumor proliferation of A375 melanoma cells in nude mice. (P) BGN in CAFs promote local metastasis of A375 melanoma cells in nude mice. Scale bar = 100µm. Data are shown as means ± S.D. *, **, and *** means *p* < 0.05, *p* < 0.01, and *p* < 0.001, respectively.

Above results revealed preferential BGN expression in fibroblast, or to be specific, CAFs in melanoma tissue (Figure 5A). We observed that, compared with NFs, CAFs isolated from melanoma lesions exhibited elevated expression of BGN and canonical fibroblast activation markers (FAP, α‐SMA) (Figure S4A). We also collected culture medium (CM) of CAFs and NFs and added these mediums into melanoma cells like A375 and A2058 (Figure S4B). And we found the CM collected from CAFs could promote the proliferation (Figure S4C–F), migration and invasion ability (Figure S4G–J) of melanoma cells. In order to verify the promotion effect of these processes was mediated by BGN in CAFs, we knocked down the expression of BGN in CAFs and also collected its CM then used to culture melanoma cells (Figure 5B–D). We found that the malignant behaviors of melanoma cells cultured with CM collected from shBGN CAFs group were inhibited (Figure 5E–L). Coinjected A375 melanoma cell with shNC CAFs or shBGN CAFs in nude mice subcutaneously (Figure 5M), we observed that melanoma cell with shNC CAFs group had a relatively larger tumor (Figure 5N–O and Figure S5A) and higher proliferative rate (Figure S5B,C). Furthermore, from the histopathological slides, we noticed that the local invasion like muscle infiltration was more obvious in group of melanoma cells mixed with shNC CAFs (Figure 5P). Finally, via mIHC analysis, we also observed that CAFs were scattered around the tumor bulk (Figure S5D). Therefore, we supposed that BGN positive CAFs could drives melanoma malignancy in vitro/vivo.

### 2.6 BGN–MDK Axis Acts as the Executor in the Protumor Process of Cancer Associated Fibroblasts

**FIGURE 6:**
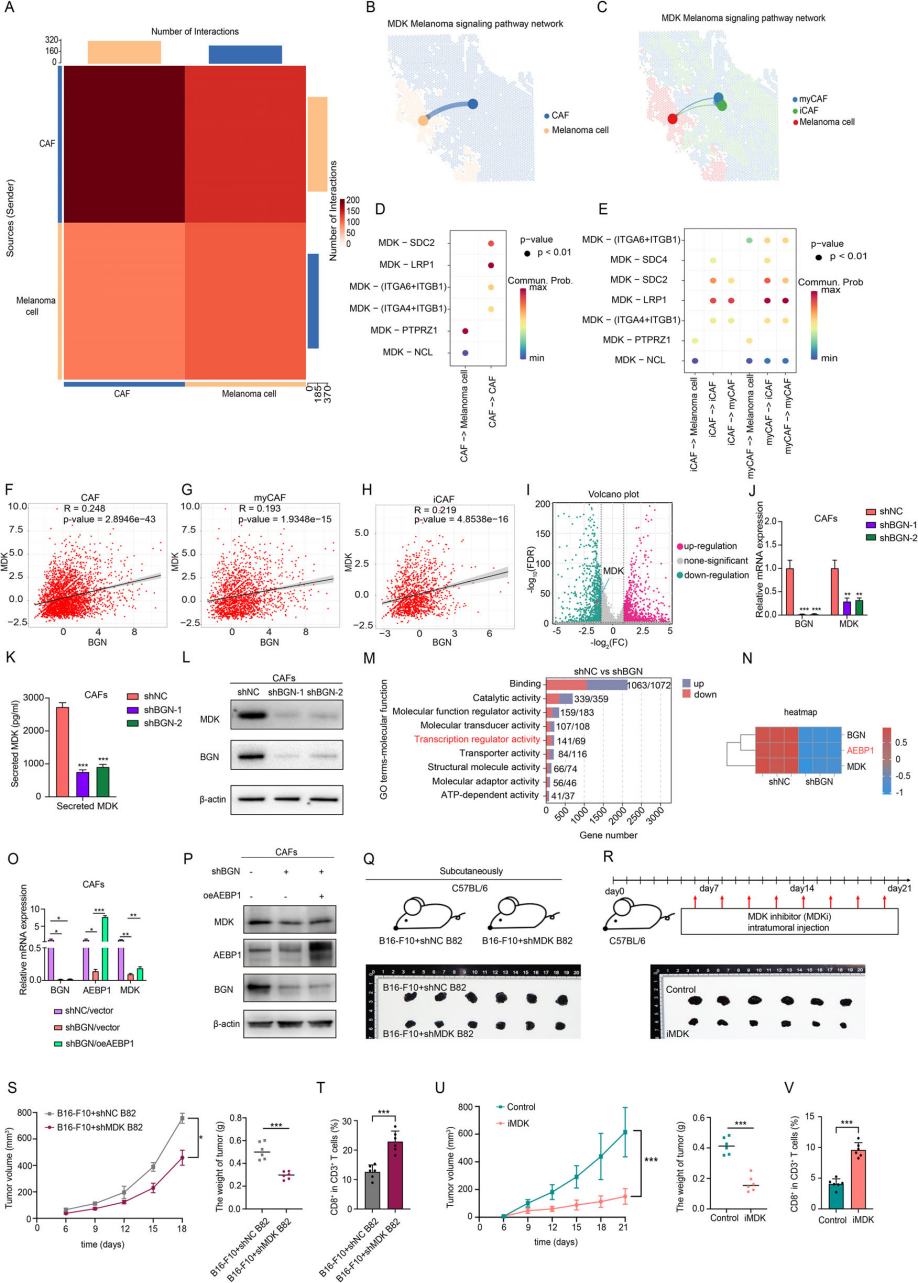
BGN–MDK axis acts as the executor in the protumor process of CAFs. (A,B) Relationship between CAFs and melanoma cells identified by cell chat analysis of spatial transcriptome. (C) Relationship between myCAFs or iCAFs and melanoma cells identified by cell chat analysis of spatial transcriptome. (D) Ligand–receptor analysis between CAFs and melanoma cells identified by cell chat analysis of spatial transcriptome. (E) Ligand–receptor analysis between myCAFs or iCAFs and melanoma cells identified by cell chat analysis of spatial transcriptome. (F–H) Relationship between MDK and BGN in CAFs, myCAFs and iCAFs from results of spatial transcriptome. (I) Volcano plot from bulk RNA‐seq of BGN‐knockdown CAFs. (J–L) Relationship between MDK and BGN of mRNA level, secreted protein level and total protein level of MDK in CAFs. (M) Gene Ontology (GO) analysis of the BGN‐knockdown transcriptome showing enrichment for terms including ‘transcription regulator activity’. (N) Heatmap from RNA sequencing of BGN‐knockdown CAFs. (O,P) MDK mRNA and protein levels in BGN‐knockdown CAFs with or without AEBP1 overexpression. (Q) Representative images of C57BL/6 mice coinjected with B16‐F10 cells and control (shNC) or MDK‐knockdown (shMDK) B82 fibroblasts. (R) Representative images in B16‐F10 tumor‐bearing C57BL/6 mice treated with vehicle or the MDK inhibitor (iMDK). (S) Tumor growth and weight of C57BL/6 mice coinjected with B16‐F10 cells and control (shNC) or MDK‐knockdown (shMDK) B82 fibroblasts. (T) Quantification of intratumoral CD8^+^ T cells in C57BL/6 mice coinjected with B16‐F10 cells and control (shNC) or MDK‐knockdown (shMDK) B82 fibroblasts. (U) Tumor growth and weight in B16‐F10 tumor‐bearing C57BL/6 mice treated with vehicle or the iMDK. (V) Quantification of intratumoral CD8^+^ T cells in in B16‐F10 tumor‐bearing C57BL/6 mice treated with vehicle or the iMDK. Data are shown as means ± S.D. *, **, and *** means *p* < 0.05, *p* < 0.01, and *p* < 0.001, respectively.

The above results hinted us that BGN positive CAFs may play a role in melanoma progression. Hence, we wanted to explore the interaction form and molecules between melanoma cell and CAFs. The cell chat analysis of spatial transcriptome showed that CAFs had connection with melanoma cells (Figure 6A,B; Figure S6A) and we also found that myCAFs had connection with melanoma cells (Figure 6C; Figure S6B,D). Cell chat analysis of published scRNA‐seq also showed us that melanoma cells had the strongest connection with fibroblasts (Figure S6C). Since the CM collected from CAFs could drives melanoma malignancy, we mainly focus on secreted signaling instead of ECM‐receptor interaction and cell contact.

Hence, we sought to identify the key secreted mediator downstream of BGN in CAFs. Cell–cell communication analysis of our spatial transcriptome and published scRNA‐seq data predicted that MDK serves as a key secreted signaling molecule between CAFs and melanoma cells (Figure 6D,E; Figure S6E–J), highlighting it as a candidate. We then examined the relationship between BGN and MDK expression. Spatial transcriptomic analysis revealed a significant positive correlation between their expression levels across all CAFs, myCAFs, and iCAFs (Figure 6F–H). Further, to obtain BGN‐regulated pathways, we performed bulk RNA sequencing (bulk RNA‐seq) following BGN knockdown in CAFs. This analysis confirmed MDK as one of the most significantly downregulated genes, providing transcriptome support for its regulation by BGN (Figure 6I). Finally, to establish direct causal regulation, we manipulated BGN expression in CAFs. BGN knockdown decreased MDK expression at the transcriptional, secretory, and total protein levels (Figure 6J–L), definitively establishing BGN as an upstream regulator of MDK. Collectively, these results showed that MDK could serve as a key secreted factor linking BGN to intercellular communication in the melanoma microenvironment.

We next sought to elucidate the mechanism by which BGN regulates MDK transcription. Gene Ontology analysis of the BGN‐knockdown bulk RNA‐seq in CAFs highlighted ‘transcription regulator activity’ as one of top enriched terms (Figure 6M). Within this category, the transcriptional regulator AEBP1, which was also downregulated in the BGN‐knockdown CAFs (Figure 6N), attracted our attention because its expression correlated positively with both BGN and MDK across all CAF subtypes in our spatial transcriptomic data (Figure S7A). To test if AEBP1 mediates BGN's effect, we performed a rescue experiment. Restoring AEBP1 expression in BGN‐knockdown CAFs significantly reversed the downregulation of both MDK mRNA and protein (Figure 6O‐P). These results suggest that BGN modulates MDK expression, at least in part, through an AEBP1‐dependent transcriptional pathway.

To functionally validate MDK as the protumorigenic effector downstream of BGN, we engineered three lentiviral‐transduced CAF cohorts: (1) shNC/vector, (2) shBGN/vector, (3) shBGN/oeMDK (Figure S8A–C). CM derived from these groups were applied to A375/A2058 melanoma cells for functional assessment. After reversing MDK expression in CAFs, its CM could promote the malignant behaviors of melanoma cells compared with shBGN/vector group (Figure S8D–K). Accordingly, this confirms that MDK is a major functional output of BGN signaling in CAFs.

To evaluate the pathophysiological and therapeutic relevance of this axis within an intact tumor microenvironment, we employed immunocompetent mouse models C57BL/6 mice. Since well‐characterized mouse CAF lines are not readily available, we utilized B82 mouse dermal fibroblasts. To endow them with CAF‐like phenotype relevant to our study, B82 cells were cocultured continuously with B16‐F10 melanoma cells prior to in vivo experiments. Using this approach, we generated MDK‐knockdown B82 fibroblasts (shMDK‐B82). Coinjection of B16‐F10 melanoma cells with shMDK‐B82 cells into C57BL/6 mice resulted in slower tumor growth and increased intratumoral CD8^+^ T cell infiltration compared to controls (B16‐F10 melanoma cells with shNC‐B82 cells) (Figure 6Q,S,T), indicating that fibroblast‐derived MDK may foster an immunosuppressive niche. Furthermore, pharmacological iMDK in established B16‐F10 tumors recapitulated these effects, suppressing tumor growth and enhancing CD8^+^ T cell infiltration (Figure 6R,U,V).

In summary, we delineate a BGN–MDK signaling axis in CAFs: BGN promotes MDK transcription via AEBP1, and the secreted MDK subsequently drives melanoma progression and may influence CD8^+^ T cell infiltration. This axis represents a promising therapeutic target, as evidenced by the efficacy of MDK inhibition.

### 2.7 BGN–MDK Axis Also Involved in the Process of Normal Fibroblast Activation in Melanoma Tumor Microenvironment

**FIGURE 7:**
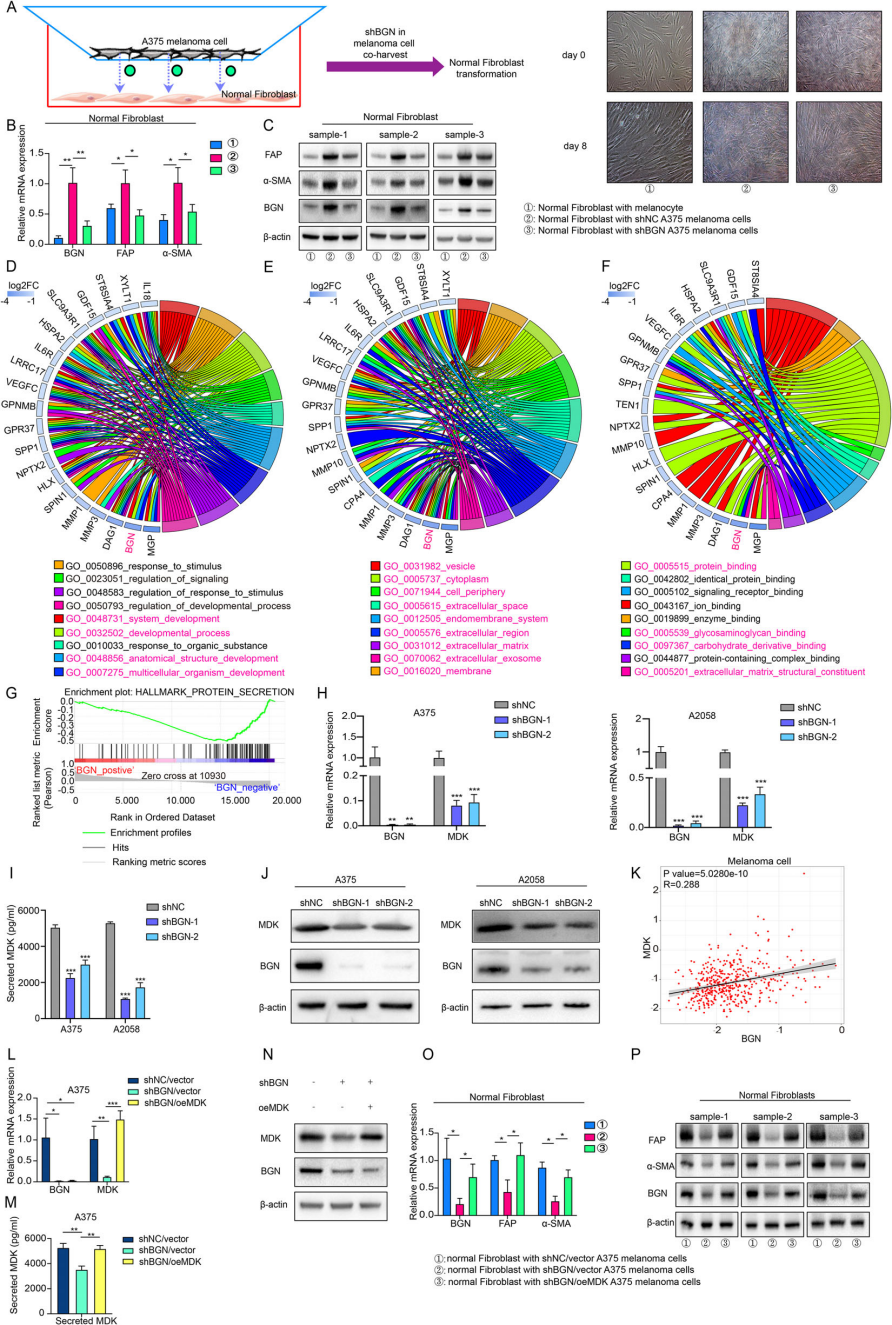
BGN–MDK axis involved in the process of NFs activation in melanoma tumor microenvironment. (A) The schematic diagram of coculture model. (B,C) The RNA and protein level of BGN, FAP, and α‐SMA in NFs cocultured with shNC A375 melanoma cells after 8 days. (D–F) GO analysis of differentially expressed genes in shNC and shBGN A375 melanoma cells from bulk RNA‐seq. (G) Potential function of BGN revealed by GSEA analysis. (H–J) Relationship between MDK and BGN of mRNA level, secreted protein level and total protein level of MDK in A375 and A2058 melanoma cells. (K) Relationship between MDK and BGN in melanoma cells from results of spatial transcriptome. (L–N) In BGN‐downregulation A375 melanoma cells, MDK was overexpressed. mRNA, secreted protein and total protein expression level of MDK were detected by RT‐qPCR, ELISA and western blot. (O,P) The RNA and protein level of BGN, FAP, and α‐SMA in NFs cocultured with BGN shNC or shBGN with vector or MDK overexpressed A375 melanoma cells after 8 days. Data are shown as means ± S.D. *, **, and *** means *p* < 0.05, *p* < 0.01, and *p* < 0.001, respectively.

Lastly, we also wonder if melanoma cells could reversely have an influence on the activation of NFs, which could be the soil for the progression of melanoma cells. Via in vitro co‐culture models, we cocultured the A375 melanoma cells (shNC and shBGN) with NFs (Figure 7A). After 8 days, we collected the RNA and protein of NFs and noticed that the RNA and protein level of BGN, FAP, and α‐SMA were up‐regulated in NFs cocultured with shNC A375 melanoma cells (Figure 7B,C). Furthermore, we also noticed BGN was involved in various processes related with protein binding and ECM activity in A375 melanoma cells from bulk RNA‐seq (Figure 7D–G). Since MDK may be involved in the interaction from melanoma cells to fibroblast, we also verified that BGN expression level has a correlation with the MDK expression at RNA (Figure 7H) and protein levels (Figure 7I,J) and from the spatial transcriptome results (Figure 7K). Similar with above methods, we also transfected vector lentivirus to the shNC and shBGN A375 melanoma cells, and transfected lentivirus of MDK overexpression to shBGN A375 melanoma cells (Figure 7L–N). We found that the expression of BGN, FAP, and α‐SMA were reversed (Figure 7O,P). Therefore, these results indicate that the BGN–MDK axis can drive the functional activation of NFs toward a CAF‐like phenotype.

## 3 Discussion

Melanoma, a highly aggressive skin cancer originating from malignant melanocyte transformation, exhibits distinct epidemiological patterns: cutaneous melanoma predominates in Caucasians, whereas acral melanoma is the predominant subtype in Asian populations [23]. Despite improved melanoma mortality rates, it remains a critical therapeutic challenge demanding urgent intervention [24].

m^6^A represents the predominant RNA modification in eukaryotic mRNA [25]. The m^6^A regulatory axis, comprising writers (methyltransferases), erasers (demethylases), and readers (recognition proteins), orchestrates RNA metabolism (transport, translation, degradation) to modulate cellular biological process [26]. Currently, numerous studies have indicated that certain proteins modified by m^6^A modification can regulate the biological behaviors of melanoma, primarily by affecting the melanoma cells themselves. For instance, our previous research found that the m^6^A methylation recognition protein YTHDF3 can regulate the translation process of LOXL3 mRNA in melanoma cells by affecting the translation initiation factor eIF3A, thereby controlling the invasion and metastasis of melanoma [4]. However, some literatures noticed that YTHDF3 was not a mere cooperator of YTHDF1, it could serve as an independent element in RNA modification [27, 28, 29, 30, 31]. As a type of m^6^A methylation reader protein with extensive regulatory functions, YTHDF3 dually governs target mRNA translation efficiency and stability [28, 32]. A plenty of previous research showed that YTHDF3 influence the process of translation of its targeted RNA without affecting its RNA level like YTHDF1 [32, 33]. However, some researchers pointed out that YTHDF3 could also influence the stability of m^6^A‐modified RNA [28, 30]. Interestingly, after interfering with the expression of YTHDF3 in melanoma A375 cells, we discovered a type of glycoprotein, namely BGN could have altered RNA levels. And its mRNA could bind to YTHDF3. BGN, an ECM structural component, is a gene‐encoded glycoprotein featuring a core protein with chondroitin sulfate/dermatan sulfate (CS/DS) glycosaminoglycan (GAG) chains [34]. Furthermore, various researches have pointed out that BGN could exert an oncogenic role in tumorgenesis. For instance, breast cancer patients exhibiting elevated levels of BGN generally had poorer distant metastasis‐free survival outcomes [35]. BGN exhibited compartment‐specific enrichment, with significantly higher expression in tumor stroma versus epithelial regions [35]. BGN knockdown suppressed lung metastasis, attenuated tumor angiogenesis, enhanced CD8^+^ T‐cell infiltration, and promoted vascular normalization through TNF‐α/Ang2 signaling downregulation [35]. BGN reconstitution in HER‐2^+^/BGNˡ°ʷ cells elevated MHC‐I expression and suppressed miR‐21‐3p, directly countering MHC‐I‐mediated immune evasion [36]. This interaction not only stimulated cell proliferation but also enhanced the production of proinvasive growth factors, such as TGF‐β [36]. Simultaneously, it suppressed the expression of immune‐stimulatory molecules and extracellular matrix components, including BGN [36]. We found that the YTHDF3 could not only protect the stability of BGN but promote the process of its translation initiation. Therefore, we provide some evidence that YTHDF3 has its dual role in regulating its targeted RNAs. However, since the m^6^A modification network is dynamic, reversible, and interactive, we further explore the regulatory roles of other potential molecules in the expression process of BGN, such as METTL14 and YTHDC1. Our combinatorial knockdown experiments revealed that YTHDF3, METTL14, and YTHDC1 collectively but independently contribute to sustaining high BGN expression levels, forming a parallel, nonhierarchical regulatory network. Unlike YTHDF3 mainly located in cytoplasm, METTL14 and YTHDC1 were located in nucleus and mainly affecting the RNA level of its targets like BGN. This spatial distinction—nuclear METTL14/YTHDC1 and cytoplasmic YTHDF3—supports a model of compartmentalized regulation, where each factor modulates BGN within its primary subcellular domain, yet their effects may converge on the same transcript. The core integrative node of this network is the m^6^A modification itself, as demonstrated by our targeted demethylation experiment. Specific removal of m^6^A from BGN mRNA abolished reader binding and reduced its expression, providing direct evidence that the m^6^A site is essential for the coordinated regulation by these factors. Since above introduced process is an intracellular process in melanoma cells, we hence explored the expression pattern and biological function of BGN in melanoma cell. Same as reported literatures in other tumors, BGN also played an oncogenic role in melanoma progression. Clinically, melanoma progression from horizontal to vertical growth phases intensifies metastatic aggressiveness. Tumor dissemination capacity is further modulated by microenvironmental constituents, particularly cancer‐associated stromal cells. Besides, we noticed that BGN was not only expressed in tumor cells in melanoma tissue slides, it also had a high expression in other cells such as fibroblasts. Hence, we wondered if fibroblast was involved in this process.

As primary constituents of dermal stroma, fibroblasts maintain tissue homeostasis [37], Within tumor microenvironments, these transform into CAFs, actively promoting tumor progression and therapy resistance [38, 39, 40]. Single‐cell transcriptomics resolves heterogeneous CAF populations within TME [41]. Fibroblast predominance within skin stroma positions CAFs expansion as a pivotal driver of melanoma progression. Conserved across malignancies, BGN^+^ CAFs demonstrate potent oncogenicity—exemplified in triple‐negative breast cancer (TNBC) where CAF‐specific BGN overexpression orchestrates immunosuppressive TME programming through TGF‐β/CXCL12 upregulation, directly excludes CD8^+^ T‐cell infiltration, and predicts adverse clinical outcomes [42]. Same results were also observed in pan‐cancer cohorts [43]. BGN overexpression in gastric cancer (GC) tissues versus adjacent normal compartments drives tumor progression and orchestrates mesothelial cell (MC) conversion into cancer‐associated fibroblast‐like cells (CAFLCs). Mechanistically, GC‐secreted BGN engages TLR2/4 receptors on MCs, activating NF‐κB signaling to induce CAFLC transformation [44]. We definitely found that BGN was markedly upregulated in melanoma CAFs. However, our mechanistic investigation revealed a paradigm distinct from above canonical paracrine/receptor‐mediated signaling. In our model, BGN served primarily as an intracellular regulator within CAFs, not as a secreted ligand engaging autocrine or paracrine TLR2/4 loops. Besides, we did not observe enrichment of above‐mentioned classical NF‐κB pathways in our bulk RNA‐seq of CAFs with or without BGN downregulation. Instead, combined the results of bulk RNA‐seq and spatial transcriptomic data, we identified AEBP1 as the critical downstream transcriptional mediator, establishing a novel BGN/AEBP1/MDK axis that drives oncogenic communication within the melanoma TME.

Since myCAFs and iCAFs were the main elements of CAFs, we also preliminarily investigated the relationship between BGN and these two subgroups. It seemed that BGN had a slightly higher expression in myCAFs than iCAFs. The origin and transformation between myCAFs and iCAFs were still unclear. Consistent with tumor progression dynamics, iCAFs dominate early‐stage malignancies while myCAFs prevail in advanced disease stage [45]. Our cell trajectory analysis showed that iCAFs could evolved into myCAFs with increasing expression of BGN. Therefore, it's rationale to suppose that the accumulation of BGN positive myCAFs in tumor microenvironment may be associated with melanoma progression.

Although it's thrilled to arrive at the above conjecture, it's still hard for us to sort the myCAFs and iCAFs due to the technology and inconsistent markers. Hence, we sorted the fibroblasts from different melanoma tissues and assumed these cells as CAFs. Only two traditional widely used pan‐CAFs markers like FAP and α‐SMA were applied to ascertain these cells. Our results supported our hypothesis that CAFs could promote melanoma progression via BGN. Since above processes was verified by cell culture medium and BGN was a main extracellular protein, we further want to know if some secreted proteins were involved in this process. There were several secreted proteins identified by cell chat analysis, while we mainly focused on MDK. Reasons could boil down to three points. First, it was a secreted oncogenic protein which was reported in several studies. Within gastric cancer peritoneal metastatic niches, CAFs display marked upregulation of estrogen receptor (ER) and MDK [46]. These fibroblasts could interact with tumor cells via above signaling pathway [46]. And estrogen stimulation could promote ovarian fibroblasts to secrete MDK, which in turn bound to LRP1, facilitating malignancy of GC cells [46]. Melanoma‐secreted MDK establishes an immune‐evasive microenvironment, correlating with poor clinical outcomes and immune checkpoint inhibitor resistance. Mechanistically, MDK orchestrates transcriptional reprogramming in melanoma cells, concurrently activating NF‐κB while suppressing interferon‐response pathways [47]. This MDK‐driven secretome subsequently educated macrophages to adopt tolerant phenotypes, which in turn led to CD8^+^ T cell dysfunction [47]. Schwann cells drive aggressive phenotypic shifts in pancreatic ductal adenocarcinoma (PDAC), promoting basal‐like tumor cell states and iCAF differentiation through paracrine signaling [48]. Schwann cells codrive PDAC progression through dual signaling: MDK‐mediated tumor cell proliferation/migration and IL‐1α‐dependent iCAF differentiation [48]. Second, the RNA and protein expression level of MDK could be associated with BGN. We verified this in different dimensions: correlation analysis from spatial transcriptomics and expression level of these two molecules in CAFs derived from melanoma tissues. Furthermore, we also elucidated a mechanistic link in CAFs. BGN regulates MDK expression, at least in part, through modulating the transcriptional regulator AEBP1. Rescue experiments confirmed that restoring AEBP1 in BGN‐deficient CAFs reversed MDK downregulation, establishing a BGN/AEBP1/MDK axis within CAFs. Besides, to validate the functional impact of this stromal axis in an immune relevant context and to assess its therapeutic potential, we utilized immunocompetent C57BL/6 mouse models. These in vivo investigations demonstrated that targeting the BGN–MDK axis in CAFs or pharmacological inhibition of MDK both significantly impairs tumor progression and may influence the CD8^+^ T cell infiltration. This finding not only confirms the central role of this axis in driving melanoma progression but also highlights its dual function in mediating immune regulation. The pharmacological validation in this study supports the BGN–MDK axis as a novel and actionable therapeutic target. Collectively, this evidence suggests that disrupting this axis may hold promise for simultaneously undermining the tumor's growth drive and affecting CD8^+^ Tcell infiltration, providing a new rationale and experimental basis for developing combination strategies targeting the tumor microenvironment. Finally, according to our results, spatial transcriptomics with CellChat analysis indicates MDK mediates signal transmission from CAFs to melanoma cells. Published single‐cell RNA sequencing studies further reveal MDK‐driven bidirectional communication between fibroblasts and melanoma cells. This may help us further explain the interactive loop between CAFs and melanoma cells and if fibroblasts could be affected and transformed by melanoma cells.

Prior research has demonstrated that secreted cytokines from tumor cells may stimulate the activation of normal fibroblasts toward a CAF‐like phenotype. In breast cancer, ligand–receptor interactions mediated by Jagged1/Notch2 within epithelial–stromal crosstalk could stimulate NF‐to‐CAF differentiation [7]. Furthermore, evidence indicated that exosomal transfer of lncRNA POU3F3 from esophageal squamous cell carcinoma (ESCC) cells to NFs subsequently induces fibroblast activation [49]. These activated fibroblasts reciprocally promoted ESCC cell proliferation and conferred cisplatin resistance through interleukin‐6 (IL‐6) secretion [49]. These studies suggested that the process of transformation of NFs can be mediated by multiple steps and was closely related to tumor cells. Therefore, it is crucial to elucidate whether and when NFs in the tumor microenvironment are regulated by tumor cells during the multistep process of tumorigenesis, leading to their activation into CAFs phenotype. Nevertheless, no substances have been found that can act as key molecules to mediate the functional interactions among melanoma cells, NFs, and CAFs, thereby promoting the progression of melanoma. Therefore, we speculated whether BGN also affected the fate of NFs and the origin of CAFs and at its colonization site. As mentioned above, MDK also participated in the communication process in melanoma cells to fibroblasts. A same expression pattern between BGN and MDK was also observed in melanoma cell and BGN–MDK axis could promote the fibroblast activation, namely, NFs to CAFs‐like phenotype. Therefore, we supposed that BGN–MDK axis was a vital pathway between melanoma cell and fibroblast.

### 3.1 Limitations

While this study provides an exploration of the BGN–MDK regulatory axis in the melanoma microenvironment, there are various limitations which also highlight directions for future research. First, although spatial transcriptomics was employed, the cohort size of limited sample and the inherent spatial resolution of the technique limited our ability to perform a robust deconvolution of CAF heterogeneity—such as distinct apCAF, tCAF, vCAF, and meCAF—or to conduct an in‐depth investigation of potential subtype‐specific associations (e.g., cutaneous vs. acral melanoma). Second, functional validations and mechanism exploration in this study primarily relied on established cell lines and mouse models, which may constrain the direct clinical translatability of the findings. Third, the potential transition from iCAFs to myCAFs was not dynamically validated in vivo through direct lineage tracing experiments due to current technical constraints. Lastly, although our preliminary exploration of tumor infiltrating lymphocytes was limited by the MHC mismatch between B82 and C57BL/6—rendering the altered CD8^+^ T cell infiltration strictly phenotypic—our consistent findings utilizing BGN‐knockdown human CAFs in nude mice and MDK‐knockdown B82 cells in C57BL/6 mice partially support MDK as a pro‐tumorigenic factor, further validated by the BGN/MDK regulatory axis. Future studies should aim to overcome these above limitations by expanding cohorts for spatial multiomics analyses, developing patient‐derived organoid coculture systems with CAFs, integrating spatial proteomics with in vivo technologies, and utilizing perfectly syngeneic models in tumor immunity research. These approaches will enable a more precise mapping of the CAF heterogeneity landscape.

## 4 Conclusion

Collectively, our study delineates a multifaceted and reciprocal role for the BGN–MDK axis in melanoma. Within melanoma cells, m^6^A‐mediated upregulation of BGN contributes to tumor progression and leads to the secretion MDK, which can lead to activate NFs into a CAFs‐like phenotype. Within these activated CAFs, we define a self‐reinforcing BGN/AEBP1/MDK axis, where BGN transcriptionally upregulates MDK secretion via AEBP1. This stromal axis potently fuels melanoma advancement and affects CD8^+^ T cell infiltration in melanoma TME. Thus, BGN operates as a central orchestrator in a vicious cycle: it is involved in melanoma cell‐driven fibroblast activation, and its expression in CAFs, through the AEBP1–MDK pathway, perpetuates a tumor‐supportive microenvironment. Targeting this axis, particularly in CAFs, presents a promising strategy to disrupt tumor‐stroma co–evolution and improve therapy.

## 5 Methods

### 5.1 Ethic Approval

All clinical specimens utilized in this research were acquired with informed consent, following protocols approved by the Ethics Committees of the Hospital for Skin Diseases, Institute of Dermatology, Chinese Academy of Medical Sciences & Peking Union Medical College. This study complied with the Declaration of Helsinki under approval codes 2024‐KY‐058 and 2024‐LC‐080. Animal experiments strictly adhered to institutional guidelines authorized by the same institution (approvals codes: 2024‐DW‐034, 2024‐DW‐047).

### 5.2 Tissue Samples Collection

All human specimens were obtained from our hospitals. Informed consent was obtained from all participants, including patients and healthy donors contributing clinical specimens to this investigation. CAFs were isolated from melanoma samples, which were also used for spatial transcriptomics, haematoxylin and eosin (H&E) staining, immunohistochemistry (IHC) staining, and multiplex immunohistochemical (mIHC) staining. Samples of benign nevi were used for H&E staining and IHC staining. Samples of normal healthy skin were used for isolating NFs.

### 5.3 Cell Culture

Our research group followed established protocols to isolate epidermal melanocytes from human samples, which was maintained in MELM medium containing 10% fetal bovine serum (FBS) and 1% penicillin–streptomycin. As previously reported [4], melanoma cell lines preserved in our research group such as A375 (RRID: CVCL_0132), A875 (RRID: CVCL_4733), SK‐MEL‐28 (RRID: CVCL_0526), M14 (RRID: CVCL_1395), MV3 (RRID: CVCL_W280), SK‐MEL‐5 (RRID: CVCL_0527), A2058 (RRID: CVCL_1059), B16‐F10 (RRID: CVCL_0159), B82 (RRID: CVCL_4114) were cultured under standardized conditions (37°C, 5% CO_2_). Specially, The A2058 melanoma cells were generously provided by Prof. Chunying Li. CAFs were isolated from different melanoma tissues, which was confirmed by several clinicians and pathologists. NFs were isolated from different healthy skin tissues which from foot or back. Both CAFs and NFs were maintained in Dulbecco's Modified Eagle Medium (DMEM) containing 10% FBS and 1% penicillin–streptomycin under standard culture conditions (37°C, 5% CO_2_).

### 5.4 Plasmid Construction and Lentiviral Transfection

Lentiviral vectors for gene knockdown or overexpression were constructed as follows. For human cells, shRNA sequences targeting YTHDF3, METTL14, YTHDC1, or BGN were cloned into GV493 vectors (hU6‑MCS‑CBh‑gcGFP‑IRES‑puromycin). Overexpression of human BGN or MDK was achieved using CV557 vector (Ubi‑MCS‑3FLAG‐SV40‑Cherry‑IRES‑neomycin) and GV492 vector (Ubi‐MCS‐3FLAG‐CBh‐gcGFP‐IRES‐puromycin) respectively. And human AEBP1 was cloned into GV703 vectors (CMV enhancer‑MCS‑3FLAG‑EF1a‑ZsGreen1‑T2A‑puromycin). The U6‐sgNC‐EF1A‐dCas13b‐FTO‐T2A‐puro‐WPRE and U6‐sgBGN‐EF1A‐dCas13b‐FTO‐T2A‐puro‐WPRE were cloned into CV950 vectors. For mouse cells, shRNA targeting MDK was also constructed in the same GV493 vector. Lentiviral particles were produced and used to transduce the respective cell lines. Human cell (A375, A2058 melanoma cells, and CAFs) were transduced with the human‑targeting constructs. Mouse B82 fibroblasts were transduced with the mouse MDK‑targeting shRNA lentivirus. Selection was initiated 72 h post‑transduction using puromycin (2 µg/mL) or G418 (1000 µg/mL) as appropriate for the resistance marker present in each vector.

### 5.5 RNA Isolation and Reverse Transcription‐Quantitative Polymerase Chain Reaction (RT‐qPCR)

Total RNA was isolated from cell samples using RNAiso Plus (Takara, Dalian, China). Extracted RNA underwent reverse transcription into cDNA with PrimeScript RT Master Mix (Takara, RR036A, Dalian, China), followed by quantitative PCR analysis on a Roche LightCycler 480 system. Gene expression levels were normalized to β‐actin and calculated via the 2^−ΔΔCt^ method. Primer sequences for target genes are listed below:

β‐actin Forward:5' GTGGCCGAGGACTTTGATTG 3';

β‐actin Reverse:5' CCTGTAACAACGCATCTCATATT 3';

BGN Forward:5' CTCCTCCTCATGCATTTCCAGC 3';

BGN Reverse:5' AGAGAACAGAAGGGAGGACTGGTC 3';

YTHDF3 Forward:5' GGTGTATTTAGTCAACCTGGGG 3';

YTHDF3 Reverse:5' AAGAGAACTAGGTGGATAGCCAT 3';

METTL14 Forward:5' AGTGCCGACAGCATTGGTG 3';

METTL14 Reverse:5' GGAGCAGAGGTATCATAGGAAGC 3';

YTHDC1 Forward:5' AACTGGTTTCTAAGCCACTGAGC 3';

YTHDC1 Reverse:5' GGAGGCACTACTTGATAGACGA 3';

MDK Forward:5' CCTGCAACTGGAAGAAGGAG 3';

MDK Reverse:5' CTGGCACTGAGCATTGTAGC 3';

FAP Forward:5' ATGAGCTTCCTCGTCCAATTCA 3';

FAP Reverse:5' AGACCACCAGAGAGCATATTTTG 3';

α‐SMA Forward:5' GAGGGAAGGTCCTAACAGCC 3';

α‐SMA Reverse:5' GCTTCACAGGATTCCCGTCT 3';

AEBP1 Forward:5' AGTGGACGCCTACGGAGAAA 3';

AEBP1 Reverse:5' GCTCGGATCTGGTTGTCCT 3';

### 5.6 RNA Binding Protein Immunoprecipitation (RIP)‐qPCR

RIP assays were performed with the Magna RIP Kit (Millipore, 17‐700). Briefly, target cells were harvested and lysed in RIP‐specific buffer. Lysates were immunoprecipitated by incubation with protein A/G magnetic beads conjugated to antibodies against relevant RNA‐binding proteins (RBPs) for 12 h at 4°C. Bead‐bound complexes were magnetically isolated, followed by six sequential washes. Coprecipitated RNA was quantified via RT‐qPCR.

### 5.7 m^6^A RNA Immunoprecipitation (MeRIP)‐qPCR

The MeRIP assay was conducted as per the manufacturer's instructions, employing the Magna MeRIP m^6^A Kit from Millipore (Catalogue No. 17‐10499). Initially, total RNA was extracted and sheared into fragments. Immunoprecipitation was then performed using A/G magnetic beads conjugated with an anti‐m^6^A antibody, with an IgG antibody serving as the control. Following elution from the beads, the immunoprecipitated RNA was purified. Finally, the enrichment of RNA fragments bearing m^6^A modifications was evaluated by RT‐qPCR analysis.

### 5.8 RNA Stability Assay

The A375 and A2058 cell lines, including shNC, shYTHDF3, shMETTL14, and shYTHDC1, received a 10 mg/mL concentration of actinomycin D. Total RNA was isolated at specified time points (0, 6, 12 h) for RT‐qPCR quantification of BGN transcript levels.

### 5.9 Western Blot

Cells were lysed in RIPA buffer containing protease and phosphatase inhibitor cocktails. Protein concentrations were quantified via BCA assay prior to sodium dodecyl sulfate‐polyacrylamide gel electrophoresis (SDS‐PAGE) separation and transfer onto PVDF membranes. After 2 h blocking with 5% BSA, membranes were incubated overnight at 4°C with primary antibodies. After incubation and a 30 min wash, membranes were exposed to HRP‐conjugated Goat Anti‐Rabbit IgG or HRP‐conjugated Goat Anti‐Mouse IgG antibodies at room temperature. The experiment utilized the following primary antibodies: anti‐β‐actin (#4970, 1:1000, Cell Signaling Technology), anti‐GAPDH (10494‐1‐AP, 1:10000, Proteintech), and anti‐Lamin B1 (12987‐1‐AP, 1:10000, Proteintech), anti‐BGN (16409‐1‐AP, 1:1000, Proteintech), anti‐MDK (ab52637, 1:1000, Abcam), anti‐FAP (ab207178, 1:1000, Abcam), anti‐α‐SMA (14395‐1‐AP, 1:5000, Proteintech), anti‐YTHDF3 (ab220161, 1:1000, Abcam), anti‐METTL14 (26158‐1‐AP, 1:1000, Proteintech), anti‐YTHDC1 (ab259990, 1:1000, Abcam), anti‐eIF3A (ab128996, 1:1000, Abcam), anti‐AEBP1 (A16340, 1:1000, Abclonal).

### 5.10 H&E Staining, IHC Staining, and mIHC Staining

Formalin‐fixed paraffin‐embedded (FFPE) sections were processed for H&E staining. mIHC employing anti‐BGN (16409‐1‐AP, Proteintech), anti‐FAP (ab207178, Abcam), and anti‐α‐SMA (14395‐1‐AP, Proteintech) antibodies was performed, with whole‐slide imaging conducted on a 3D HISTECH Pannoramic scanner series (DESK/MIDI/P250/P1000; Hungary).

### 5.11 Enzyme‐Linked Immunosorbent Assay (ELISA) Experiment

MDK concentrations in culture supernatants were quantified using a commercial ELISA kit (Human Midkine/MDK ELISA Kit; Boster Biological Technology, EK1235) following the manufacturer's protocol.

### 5.12 Proliferation Assays

Proliferative capacity of melanoma cells was evaluated through multiple assays: CCK‐8, EdU labeling (5‐Ethynyl‐2′‐deoxyuridine), and colony formation. For CCK‐8 analysis, A375 and A2058 cells under specified treatments were plated in 96‐well plates at 5000 cells per well. Following incubation in the culture medium supplemented with 10% CCK‐8 reagent (MedChemExpress, HY‐K0301) at 37°C for 60 min, absorbance readings at 450 nm were acquired via spectrophotometry, with optical density (OD) values documented at 0, 24, 48, and 72 h intervals. In terms of EdU assays, 5000 cells of A375 and A2058 with different conditions were placed in each well of 96‐well plates. Following manufacturer's guidelines, pictures were captured using EdU kits (BeyoClick EdU Cell Proliferation Kit featuring Alexa Fluor 594, C0078S). For colony formation analysis, A375 and A2058 melanoma cells (2000 cells per well) were plated in 6‐well plates under specified conditions. Following culture until macroscopic colony emergence, cells were methanol‐fixed and crystal violet‐stained prior to photographic documentation.

### 5.13 Invasion Assays

Invasion and migration capacities were assessed via Transwell chambers (Corning, 24‐well, 8 µm pores) and wound healing assays. For Transwell migration, 5 × 10^4^ cells in serum‐free DMEM (0.2 mL) were seeded in upper chambers, with lower chambers containing 20% FBS–DMEM (0.6 mL). Invasion assays employed Matrigel‐coated membranes under identical conditions. After 24–36 h incubation, membranes were methanol‐fixed, crystal violet‐stained, and imaged. In parallel, wound healing assays utilized confluent monolayers of 5 × 10^5^ cells per well in 6‐well plates. Linear scratches generated with 200 µL pipette tips were incubated in serum‐free DMEM, with wound closure documented at 0, 24, 48, and 72 h.

### 5.14 Coculture Assay

To generate conditioned culture medium (CM), CAFs or NFs were seeded at a standardized cell number (approximately 1 × 10^7^ cells per 10 mL of medium) in complete growth medium. Upon reaching confluence, cells were washed twice with phosphate‐buffered saline (PBS) and subsequently incubated in serum‐free and antibiotic‐free DMEM for 3 days. The collected CM was then centrifuged until use. For functional assays targeting melanoma cells, the CM was supplemented with 10% FBS and 1% penicillin–streptomycin. A375 and A2058 melanoma cells were continuously cultured in this CM‐supplemented medium for a period of 7 days prior to the assessment of malignant behaviors. In terms of coculture of melanoma cells and NFs, a 6‐well transwell chamber system with a pore size of 1.0 µm from Corning, USA, was used. A total of 1 × 10^5^ A375 cells, with or without BGN downregulation, were placed in 2 mL of medium in the upper chamber, and 1 × 10^5^ NFs were placed in the lower chamber for a coculture period of 8 days. The coculture of B16‐F10 cell and B82 cell was also employed above method.

### 5.15 In Vivo Assay

All animal procedures adhered to institutional guidelines using 5‐week‐old BALB/c nude mice or 5‐week‐old C57BL/6 mice. In terms of BALB/c nude mice experiments, 2 × 10^6^ A375 cells (shNC or shBGN) in 100 µL PBS were subcutaneously injected and monitored for 3 weeks. A suspension of 2 × 10^6^ A375 melanoma cells and 2 × 10^6^ shNC or shBGN CAFs in 100 µL of PBS was injected subcutaneously over a period of 3 weeks. shNC/shBGN cells (1.5 × 10^6^ in 100 µL PBS) were intravenously injected via tail vein and monitored over 6 weeks. Metastasis were quantified using IVIS Spectrum imaging (PerkinElmer; auto‐exposure), with radiance intensity expressed as p/sec/cm^2^/sr. In terms of C57BL/6 mice experiment, a suspension of 3 × 10^5^ B16‐F10 melanoma cells and 3 × 10^5^ shNC or shMDK B82 in 100 µL of PBS was injected subcutaneously over a period of 18 days. Besides, to evaluate the therapeutic potential of MDK inhibition, C57BL/6 mice bearing established B16‐F10 subcutaneous tumors were randomly assigned to treatment groups. The MDK inhibitor (iMDK) was prepared as follows: a 25 mg aliquot of iMDK was first dissolved in 5 mL of dimethyl sulfoxide (DMSO) to create a stock solution. This stock was then diluted with a mixture of 20 mL polyethylene glycol 300 (PEG300), 2.5 mL Tween‐80, and 22.5 mL double‐distilled water (ddH_2_O) to yield the final working solution for administration. The control group received an equivalent volume of the vehicle solvent mixture (DMSO/PEG300/Tween‐80/ddH_2_O) without iMDK. Mice were treated every other day via peritumoral and intratumoral injections (total 100 µL per injection) over a period of 21 days. Terminal procedures included euthanasia, fixation of subcutaneous tumors/lungs, and subsequent H&E/IHC staining.

### 5.16 Spatial Transcriptome Analysis

Freshly collected melanoma tissue was segmented into suitably sized blocks and promptly fixed in 4% formaldehyde before embedding in paraffin. The sections were affixed to slides in accordance with the 10× Genomics Protocol. Deparaffinization, H&E staining, imaging, and decross‐linking of the sections were conducted following the 10× protocol. Spatial transcriptomics utilized the 10× Genomics Visium CytAssist FFPE Kit, performing probe hybridization/release and library construction per manufacturer's protocol. FASTQ data were processed with Space Ranger (v2.0.1) and aligned to the GRCh38 human reference genome. Spatial transcriptomic analysis encompassed UMI count aggregation per barcode, followed by tissue‐overlay spot detection via image‐based foreground/background discrimination. The filtered matrix was processed in Seurat (v4.3.0) implementing SCTransform normalization with top 3000 highly variable gene (HVG) selection. Principal component analysis (PCA) dimensionality reduction was subsequently performed on log‐transformed gene‐barcode matrices of high‐variance genes. Cell clustering via graph‐based gene expression profiling utilized the FindClusters function, followed by UMAP dimensionality reduction (RunUMAP) for 2D visualization. Cluster‐specific marker genes were identified with FindAllMarkers, while differentially expressed genes (DEGs) were determined by FindMarkers (threshold: *p* < 0.05, |log_2_FC| > 0.58). Bioinformatics analyses—including Gene Ontology (GO), KEGG, trajectory inference, and CellChat—were performed in R v4.0.3.

### 5.17 Single Cell RNA Transcriptome Analysis

Our research involved collecting scRNA sequencing data from GSE215120, comprising six untreated acral melanoma samples and three untreated cutaneous melanoma samples. The CreateSeuratObject from the Seurat package (version 4.0.5) was employed to filter the sequencing data. We assessed the proportion of mitochondrial genes using the PercentageFeatureSet function, keeping only those cells with mitochondrial gene proportions below 10%. The analytical pipeline employed NormalizeData for feature scaling, followed by high‐variance gene detection via FindVariableFeatures. We conducted dimensionality reduction using the Seurat package and created an elbow plot to ascertain the appropriate dimensions of the data. Post‐PCA dimensionality reduction, unsupervised clustering was executed via Seurat's FindNeighbors/FindClusters functions, with results visualized through UMAP. Cell type annotation employed SingleR for automated scRNA‐seq classification. BGN expression patterns across clusters were extracted and visualized using ggpubr, followed by intercluster communication analysis via CellChat.

### 5.18 Bulk RNA Sequencing

RNA extracted from shNC/shBGN CAFs and shNC/shBGN A375 cells underwent poly(A)‐selected library preparation (PolyT tract mRNA isolation). Libraries were sequenced on Illumina NovaSeq 6000 platforms.

### 5.19 Tumor‐Infiltrating Lymphocyte (TIL) Isolation and Flow Cytometry Analysis

Single‐cell suspensions were generated from subcutaneously implanted murine melanoma tumors via mechanical dissociation. Briefly, excised tumors were minced with sterile scissors and subsequently dissociated by gentle grinding between the frosted ends of two sterile microscope slides in cold DMEM medium. The resulting cell mixture was pipetted repeatedly and filtered through a 40 µm cell strainer. Following centrifugation, red blood cells were lysed using Tris‐buffered ammonium chloride buffer. Cell viability was assessed by staining with the Zombie NIR Fixable Viability Kit (BioLegend Cat. No. 423105), and Fc receptors were blocked. For surface marker staining, cells were incubated with a cocktail containing the following antibodies: Brilliant Violet 510 anti‐mouse CD45 (BioLegend Cat. No. 103138), FITC anti‐mouse CD3 (BioLegend Cat. No. 100204), Alexa Fluor 700 anti‐mouse CD4 (BioLegend Cat. No. 100430), and Brilliant Violet 605 anti‐mouse CD8a (BioLegend Cat. No. 100744). After washing, cells were analyzed by flow cytometry. Live CD45^+^ leukocytes were gated, followed by identification of CD3^+^ T cells. TIL subsets were defined as CD4^+^ T cells (CD3^+^CD4^+^CD8^−^) and CD8^+^ T cells (CD3^+^CD4^−^CD8^+^).

### 5.20 Bioinformatic Analysis of BGN in Melanoma Tissues

BGN expression data curated from TCGA/GTEx/GSE98394 datasets underwent multifaceted analysis: differential expression profiling (normal/primary/metastatic groups) visualized via ggplot2 v3.4.0 boxplots with intergroup significance assessed by *t*‐tests; survival analysis incorporating OS/DFS curves for pan‐cancer cohorts, stratified by optimal expression cutoff for K–M survival analysis (log‐rank *p*‐values); Pearson correlation ranking of BGN‐associated genes in TCGA‐SKCM; pathway enrichment via GSEA v4.0.3 (significant threshold: NOM *p*‐value < 0.05, FDR < 25%).

### 5.21 Statistical Analysis

All experiments were conducted in triplicate. Intergroup differences were assessed by Student's *t*‐test (two groups) or ANOVA (≥3 groups), with significance denoted as: *p < 0.05, **p < 0.01, *p < 0.001; ns: nonsignificant (*p* > 0.05).

## Author Contributions

H.‐z.S. and M.‐y.W wrote the manuscript. H.‐z.S., X.‐f.C, and J.‐f.S., H.C. designed the research. H.‐z.S. and M.‐y.W. performed most experiments. J.‐q.L. and L.M. performed other experiments. H.‐z.S., M.‐y.W., and J.‐q.L. analyzed the data. J.‐q.L., C.‐c.T., X.‐m.Z., Z.‐h.S., Z.‐y.X., R.‐d.Z., S.‐y.Y., L.‐m.H., Y.W., J.‐s.X., W.‐b.B. collected samples or other materials. X.‐f.C., J.‐f.S., and H.C. supervised the study. All authors read and approved the final manuscript.

## Funding

Our research was supported by the National Natural Science Foundation of China (Grant No. 82403502), the Fundamental Research Funds for the Central Universities (Grant No. 3332024086), the CAMS Innovation Fund for Medical Sciences and Clinical Translational Project (Grant No. 2023‐I2M‐CT‐B‐110) and Natural Science Foundation of Jiangsu Province (Grant No. BK20231115).

## Ethics Statement

This research received approval from the Ethics Committee of the Institute of Dermatology, the Chinese Academy of Medical Sciences, and the Peking Union Medical College (2024‐KY‐058, 2024‐LC‐080, 2024‐DW‐034, 2024‐DW‐047).

## Conflicts of Interest

The authors declare no conflicts of interest.

## Supporting information




**Supporting File**: advs74753‐sup‐0001‐SuppMat.docx

## Data Availability

The data that support the findings of this study are available from the corresponding author upon reasonable request.
