# Supplementary material for: BGN/MDK Axis in the Melanoma Tumor Microenvironment Strengthens Tumor Malignancy by Modulating Cancer Cells and Cancer‐Associated Fibroblasts Crosstalk
Source: Adv Sci (Weinh). 2026 Mar 15;13(28):e14590. doi: 10.1002/advs.202514590 (PMC13185869; doi:10.1002/advs.202514590)
Supplement: Supplementary file 1 — Supporting File: advs74753‐sup‐0001‐SuppMat.docx [file ADVS-13-e14590-s001.docx]

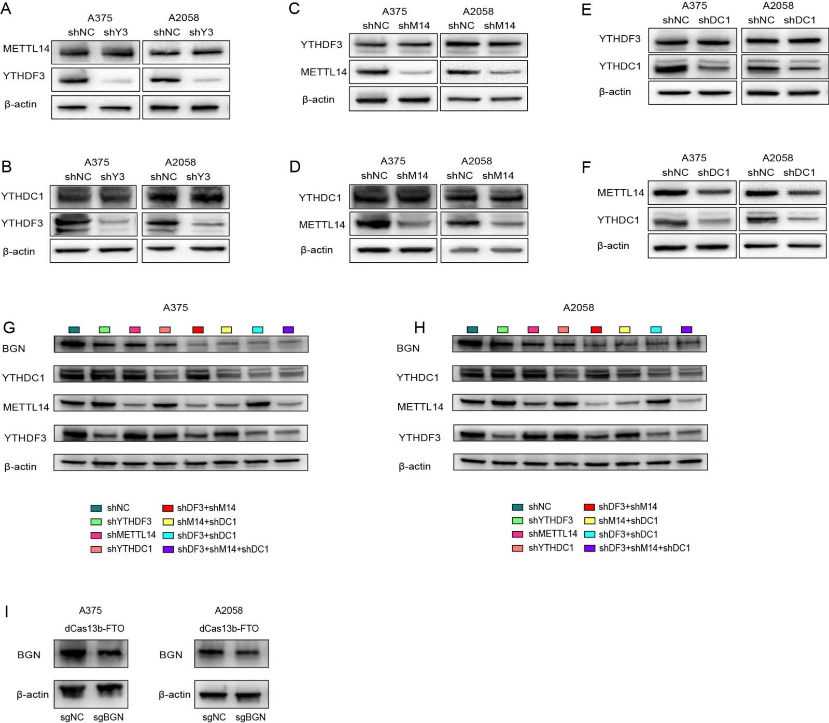


**Supplementary Figure 1 The protein expression of BGN, YTHDF3, METTL14, YTHDC1 under different conditions of YTHDF3, METTL14, YTHDC1 and m⁶A-targeting conditions.**

(A-B) Protein level of YTHDF3, METTL14 and YTHDC1 after downregulation of YTHDF3 by western blot in A375 and A2058 melanoma cells. (C-D) Protein level of METTL14, YTHDF3 and YTHDC1 after downregulation of METTL14 by western blot in A375 and A2058 melanoma cells. (E-F) Protein level of YTHDC1, YTHDF3 and METTL14 after downregulation of YTHDC1 by western blot in A375 and A2058 melanoma cells. (G-H) Protein levels of YTHDF3, METTL14, YTHDC1 and BGN after combinatorial or triple downregulation of YTHDF3, METTL14 or YTHDC1in A375 and A2058 melanoma cells measured by western blot in A375 and A2058 melanoma cells. (I) Protein levels of BGN in dCas13b-FTO-sgNC group and dCas13b-FTO-sgBGN group measured by western blot.


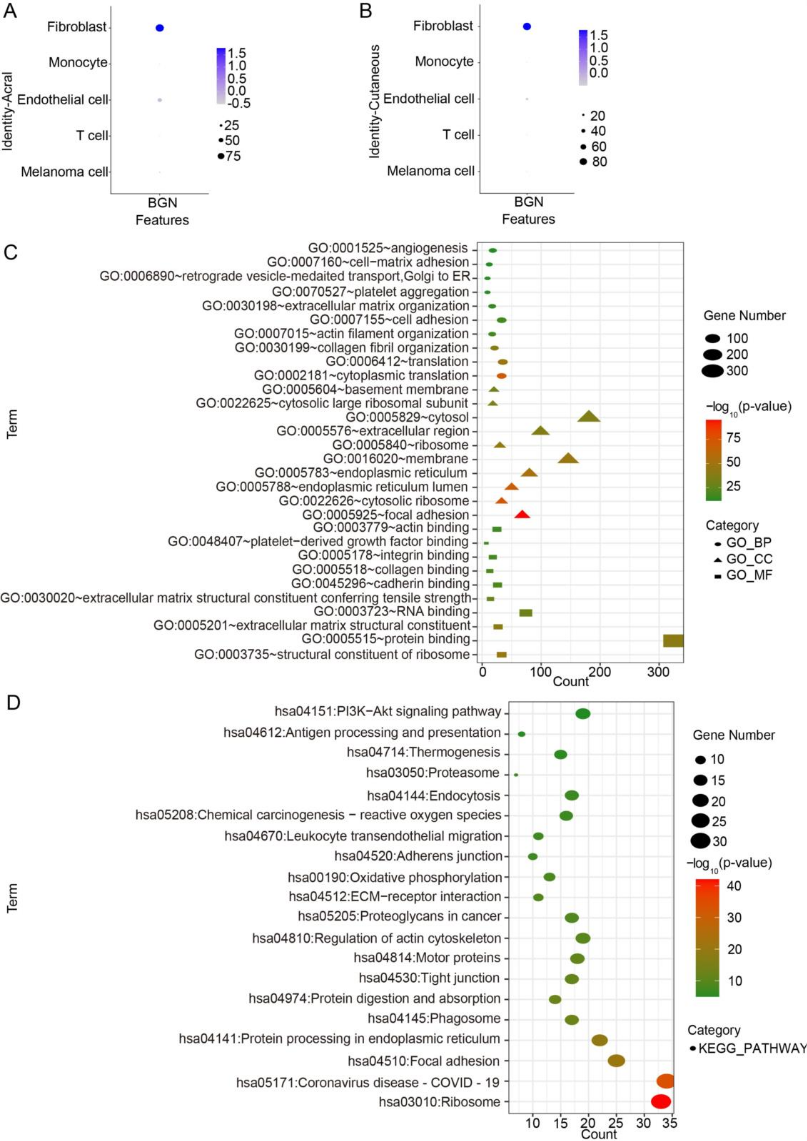


**Supplementary Figure 2 The expression of BGN in fibroblasts in melanoma tumor microenvironment from scRNA-seq data.**

(A-B) Expression of BGN from the results of scRNA-seq data in different cell types. (C-D) GO analysis and KEGG pathway analysis of BGN-downstream differentially expressed genes in fibroblasts.


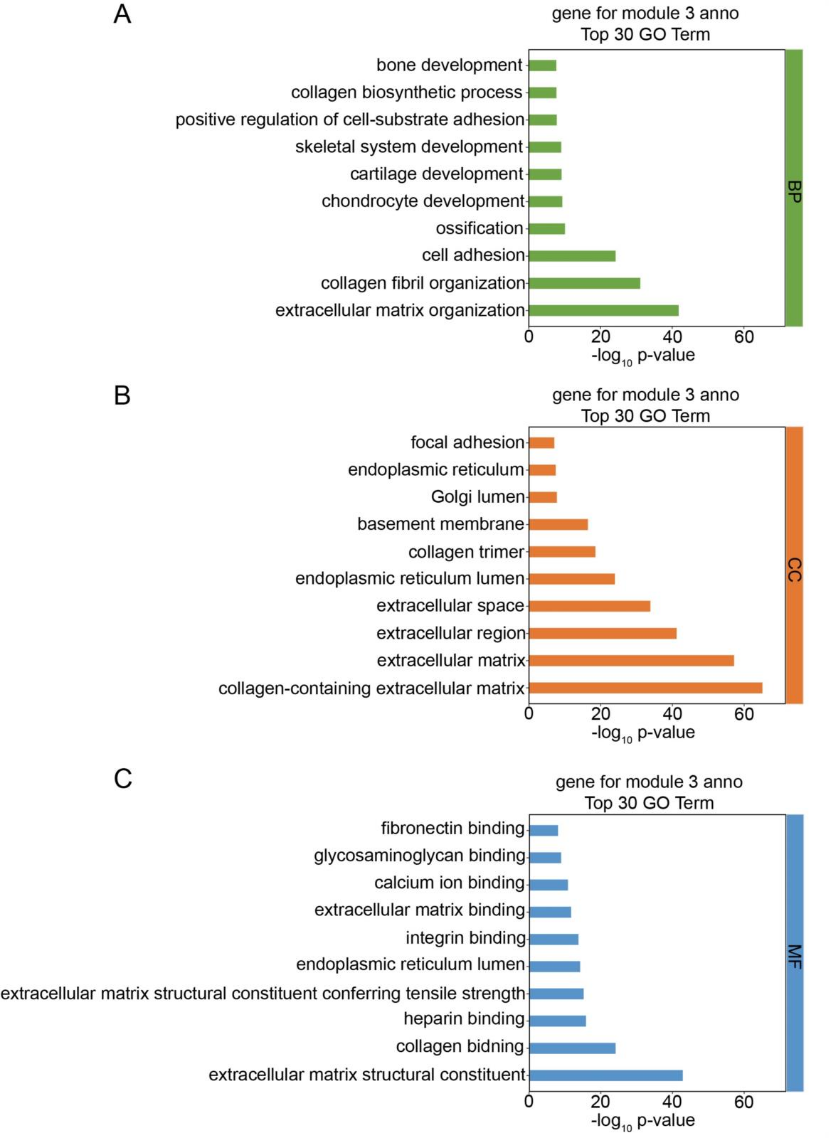


**Supplementary Figure 3 GO analysis of module 3 in evolution iCAFs to myCAFs from cell trajectory analysis.**

(A-C) The terms of GO analysis of module 3 in heatmap.


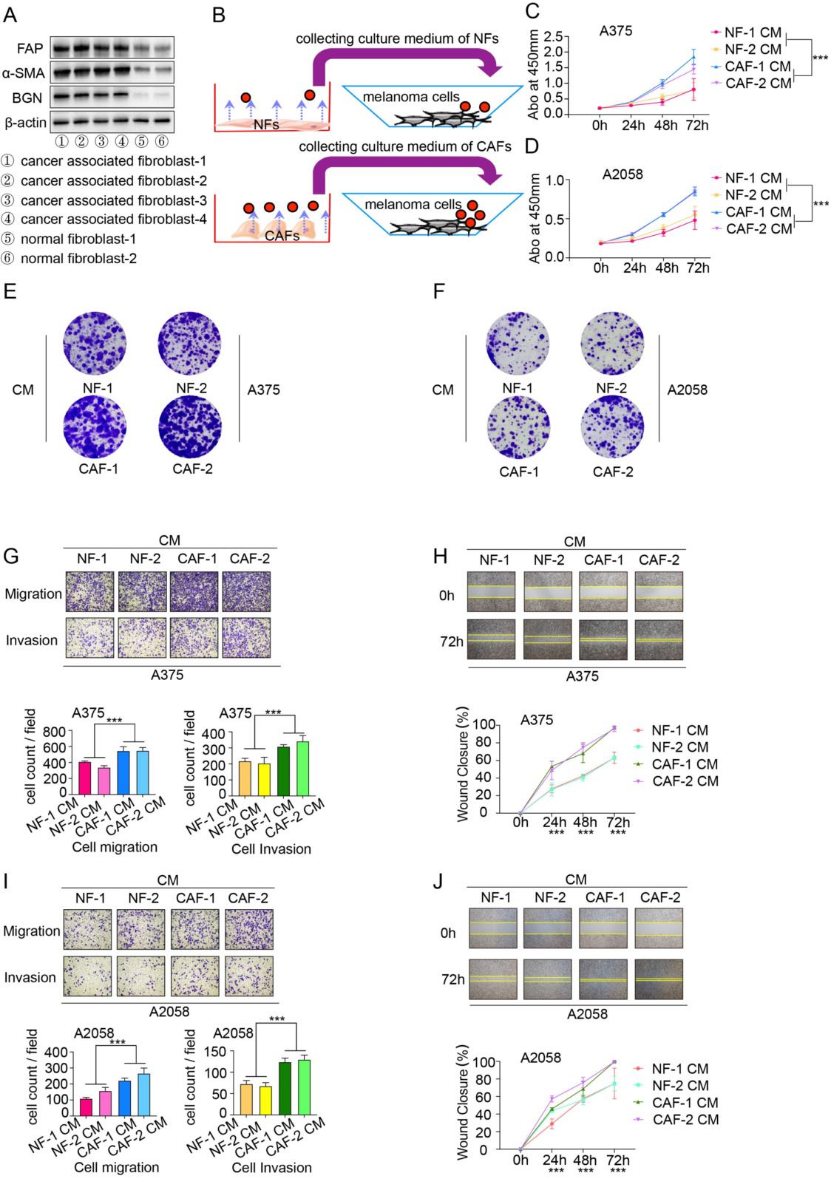


**Supplementary Figure 4 CAFs isolated from melanoma tissues plays an oncogenic role in melanoma progression.**

(A)The expression level of BGN or other proteins related with fibroblasts activation like FAP and α-SMA in CAFs isolated from melanoma tissues. (B) The schematic diagram of culture model. (C-F) CCK-8 assay and clone formation assay show that CAFs isolated from melanoma tissues derived culture medium promotes proliferation ability of A375 and A2058 melanoma cells. (G-J) Transwell assay and wound healing assay show that CAFs isolated from melanoma tissues derived culture medium promotes migration and invasion ability of A375 and A2058 melanoma cells. Data are shown as means ± S.D. *, ** and *** means *p* < 0.05, *p* < 0.01 and *p* < 0.001 respectively.


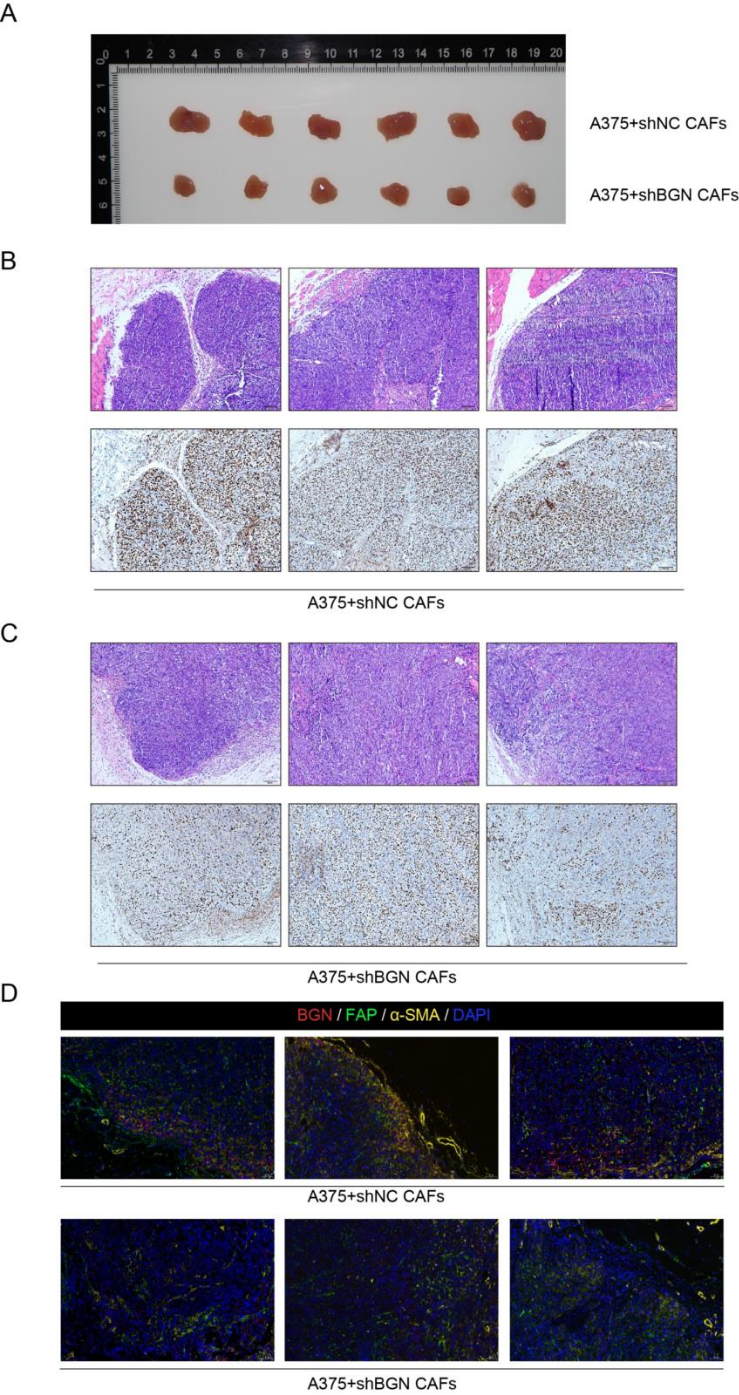


**Supplementary Figure 5 BGN in CAFs plays an oncogenic role in melanoma proliferation in vivo**

(A-C) BGN in CAFs promote subcutaneous tumor proliferation of A375 melanoma cells in nude mice. (D) The mIHC staining (BGN, FAP and α-SMA) of CAFs in subcutaneous tumor. Scale bar= 50μm.


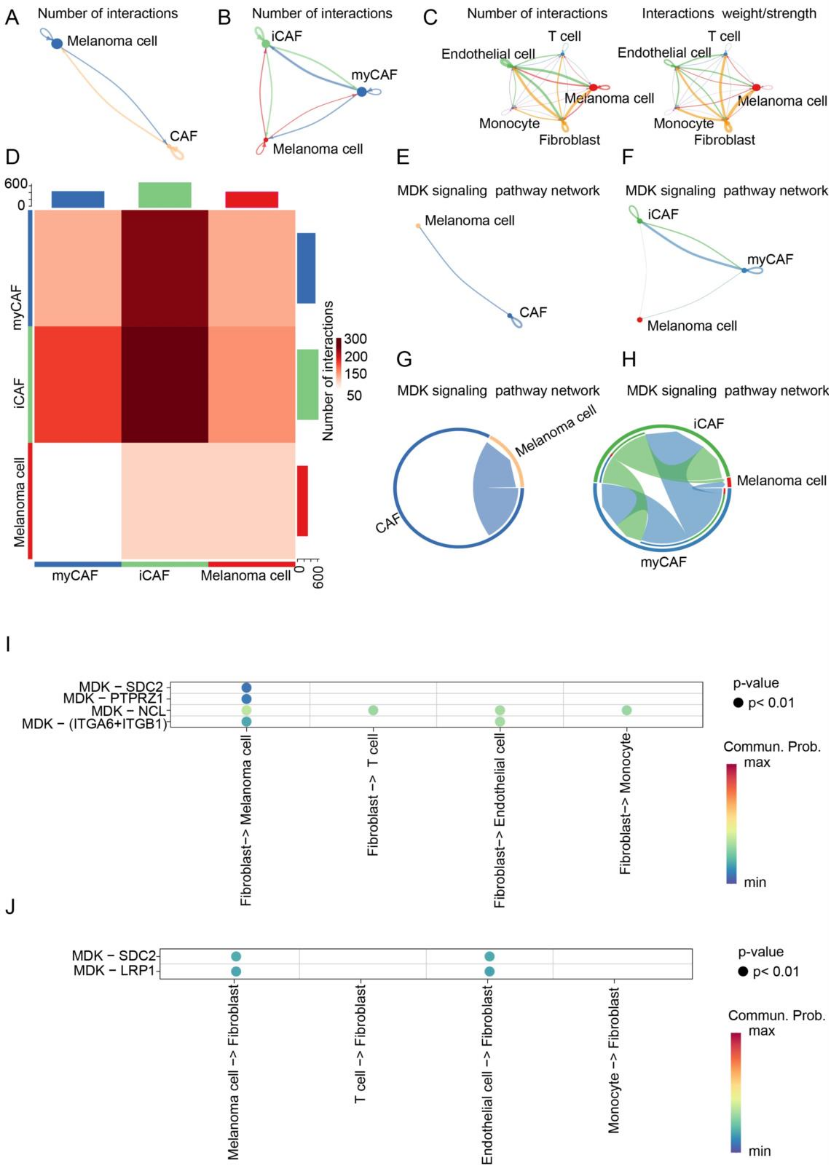


**Supplementary Figure 6 Relationship between CAFs or fibroblasts and melanoma cells identified by cell chat analysis.**

(A-B) Number of interactions between CAFs, myCAFs or iCAFs and melanoma cells identified by cell chat analysis of spatial transcriptome. (C) Number of interactions between different cell types by cell chat analysis of scRNA-seq data. (D) Heat map of interactions between myCAFs or iCAFs and melanoma cells identified by cell chat analysis of spatial transcriptome. (E-H) MDK pathway in interactions between CAFs, myCAFs or iCAFs and melanoma cells identified by cell chat analysis of spatial transcriptome. (I-J) Ligand-receptor analysis between different cell types by cell chat analysis of scRNA-seq data.


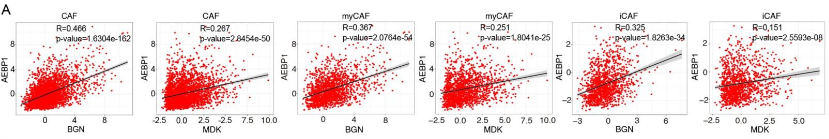


**Supplementary Figure 7 Relationship between BGN, MDK and AEBP1 in CAF, myCAF and iCAF.**


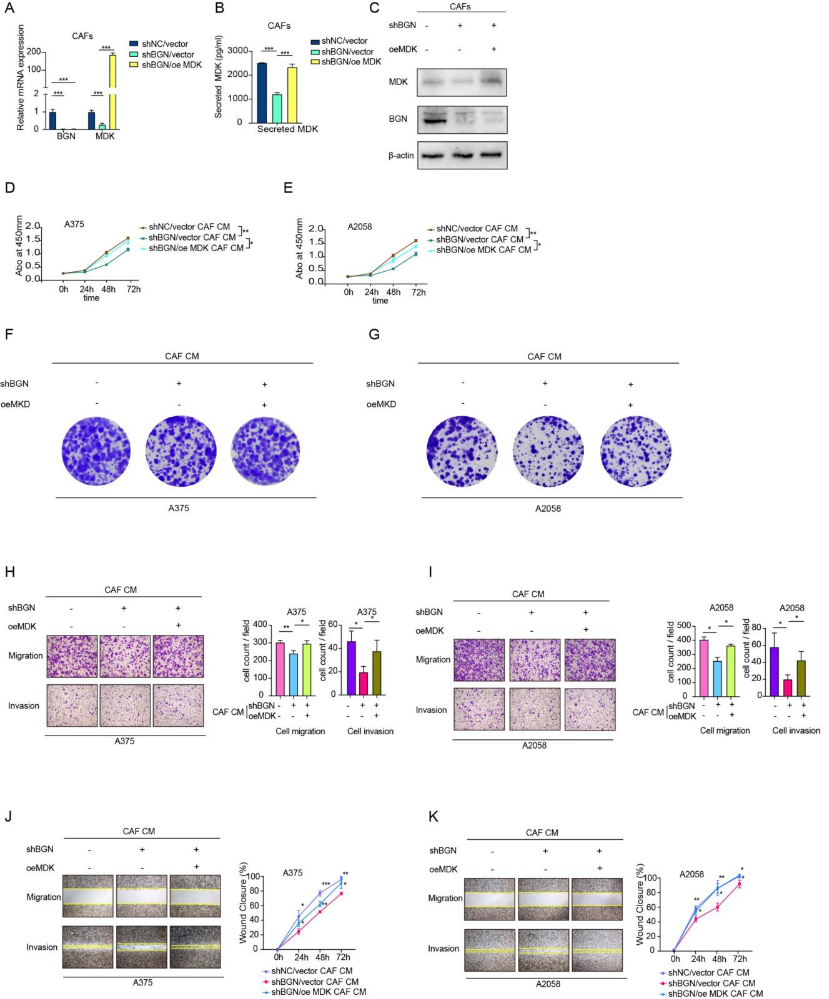


**Supplementary Figure 8 BGN-MDK axis in CAFs promotes malignant behaviors of melanoma cells.**

(A-C) In BGN shNC or shBGN with vector or MDK overexpressed CAFs, mRNA, secreted protein and total protein expression level of MDK were detected by RT-qPCR, ELISA and western blot. (D-E) CCK-8 assay shows that MDK overexpression in BGN- downregulation CAFs derived culture medium promotes proliferation ability of A375 and A2058 melanoma cells. (F-G) Clone formation assay show that MDK overexpression in BGN- downregulation CAFs derived culture medium promotes proliferation ability of A375 and A2058 melanoma cells. (H-I) Transwell assay show that MDK overexpression in BGN-downregulation CAFs derived culture medium promotes migration and invasion ability of A375 and A2058 melanoma cells. (J-K) Wound healing assay show that MDK overexpression in BGN- downregulation CAFs derived culture medium promotes migration and invasion ability of A375 and A2058 melanoma cells. Data are shown as means ± S.D. *, ** and *** means *p* < 0.05, *p* < 0.01 and *p* < 0.001 respectively.
